# Ubiquitination of NS1 Confers Differential Adaptation of Zika Virus in Mammalian Hosts and Mosquito Vectors

**DOI:** 10.1002/advs.202408024

**Published:** 2024-08-19

**Authors:** Chenxiao Huang, Tao Jiang, Wen Pan, Tingting Feng, Xia Zhou, Qihan Wu, Feng Ma, Jianfeng Dai

**Affiliations:** ^1^ Institutes of Biology and Medical Sciences MOE Key Laboratory of Geriatric Diseases and Immunology Jiangsu Key Laboratory of Infection and Immunity Soochow University Suzhou 215000 China; ^2^ Department of Clinical Laboratory The Affiliated Suzhou Hospital of Nanjing Medical University Suzhou Municipal Hospital Gusu School of Nanjing Medical University Suzhou 215000 China; ^3^ School of Biology and Basic Medical Science Suzhou Medical College of Soochow University Suzhou 215000 China; ^4^ Shanghai‐MOST Key Laboratory of Health and Disease Genomics NHC Key Lab of Reproduction Regulation Shanghai Institute for Biomedical and Pharmaceutical Technologies Shanghai 200000 China; ^5^ National Key Laboratory of Immunity and Inflammation and CAMS Key Laboratory of Synthetic Biology Regulatory Elements Suzhou Institute of Systems Medicine Chinese Academy of Medical Sciences & Peking Union Medical College Suzhou 215123 China

**Keywords:** E3 ligase WWP2, flavivirus, mosquito, NS1, ubiquitination, Zika virus

## Abstract

Arboviruses, transmitted by medical arthropods, pose a serious health threat worldwide. During viral infection, Post Translational Modifications (PTMs) are present on both host and viral proteins, regulating multiple processes of the viral lifecycle. In this study, a mammalian E3 ubiquitin ligase WWP2 (WW domain containing E3 ubiquitin ligase 2) is identified, which interacts with the NS1 protein of Zika virus (ZIKV) and mediates K63 and K48 ubiquitination of Lys 265 and Lys 284, respectively. WWP2‐mediated NS1 ubiquitination leads to NS1 degradation via the ubiquitin‐proteasome pathway, thereby inhibiting ZIKV infection in mammalian hosts. Simultaneously, it is found Su(dx), a protein highly homologous to host WWP2 in mosquitoes, is capable of ubiquitinating NS1 in mosquito cells. Unexpectedly, ubiquitination of NS1 in mosquitoes does not lead to NS1 degradation; instead, it promotes viral infection in mosquitoes. Correspondingly, the NS1 K265R mutant virus is less infectious to mosquitoes than the wild‐type (WT) virus. The above results suggest that the ubiquitination of the NS1 protein confers different adaptations of ZIKV to hosts and vectors, and more importantly, this explains why NS1 K265‐type strains have become predominantly endemic in nature. This study highlights the potential application in antiviral drug and vaccine development by targeting viral proteins' PTMs.

## Graphical abstract

ZIKV NS1 undergoes ubiquitination in both mammalian hosts and mosquito vectors. The mammalian E3 ligase WWP2 mediates the ubiquitination and degradation of NS1, thereby restricts ZIKV infection in mammalian hosts. In contrast, a mosquito homolog of hWWP2 enhances ZIKV infection in vectors, contributing to virus expansion.

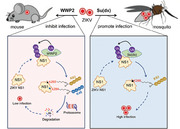

## 1 Introduction

Arboviral infections pose a severe threat to human health. Arthropod vectors, including mosquitoes and ticks, can transmit a wide range of viruses that affect humans, with notable examples being RNA viruses from the flavivirus family, such as Dengue virus (DENV), Zika Virus (ZIKV), and Japanese Encephalitis Virus (JEV).^[^ ^1^ ^]^ Currently, effective vaccines exist for a few mosquito‐borne flaviviruses, like Yellow Fever Virus (YFV) and Japanese Encephalitis Virus (JEV).^[^ ^2^ ^]^ However, safe and effective vaccines, as well as antiviral therapies, for other mosquito‐borne flaviviruses, including DENV, ZIKV, and West Nile Virus (WNV), are still lacking.

Viruses rely on host cells for various processes, including genome replication, transcription, translation, packaging, and release from infected cells. Post‐translational modifications (PTMs) of proteins play a crucial role in regulating protein localization, structure, and function.^[^ ^3^ ^]^ PTMs include adding small proteins or functional groups to specific amino acids within proteins, including ubiquitination, lipidation, glycosylation, methylation, phosphorylation, and acetylation.^[^ ^4^ ^]^ PTMs are executed by specialized enzymes such as ubiquitin ligases, glycosyltransferases, poly (adenosine diphosphate ribose) polymerase (PARP), acetyltransferases, and kinases.^[^ ^4^, ^5^ ^]^ PTMs modulate protein conformation (by altering charge or hydrophobicity), interactions, signaling transduction, or lead to degradation, playing various roles in the cell.^[^ ^6^ ^]^ In the context of viral infection, PTMs can facilitate viral infection by modifying relevant host or viral proteins to regulate viral replication, assembly, release and inhibit the interferon response. Conversely, hosts can counteract viral infection by removing PTMs critical for viral protease activity or adding small molecules such as ubiquitin or ubiquitin‐like proteins, leading to modification and subsequent inactivation or proteasome‐mediated degradation.^[^ ^3^ ^]^

A growing body of research has shown that viral‐encoded proteins undergo post‐translational modifications (PTMs) in host cells during viral infection. These PTMs regulate various stages of viral infection. For example, the host ubiquitin system modifies viral proteins and directs them for proteasomal degradation. In our previous study, we reported that the interferon‐stimulated gene (ISG) TRIM69 ubiquitinates lysine at position K104 of the DENV NS3 protein, resulting in its degradation and consequently, inhibiting viral replication.^[^ ^7^ ^]^ TRIM5α targets the NS2B3 of the tick‐borne flavivirus TBEV but does not affect mosquito‐borne flaviviruses.^[^ ^8^ ^]^ Ubiquitin‐mediated proteasomal degradation also targets the nucleoprotein (NP) of influenza A virus (IAV) and vesicular stomatitis virus (VSV), thereby inhibiting viral infection.^[^ ^9^ ^]^ The human immunodeficiency virus (HIV) integrase (IN) protein is modified by polyubiquitination toward proteasomal degradation, affecting viral replication and proviral DNA formation.^[^ ^10^ ^]^ Hepatitis C Virus (HCV) core proteins are also degraded by ubiquitination, which negatively regulates viral replication, assembly, and response to interferon‐mediated viral attacks.^[^ ^11^ ^]^In addition to ubiquitination, other protein PTMs also regulate the function of viral proteins. For example, the ubiquitin‐like protein ISG15 prevents viral RNA synthesis by ISGylation of the NP protein of IAV and inhibits its oligomerization.^[^ ^12^ ^]^SUMOylation of HIV‐1 p6 (gag protein hydrolysis product) inhibits the early viral replication process.^[^ ^13^ ^]^HDM2 E3 ligase targets IAV PB2 for Neddylation modification (K699). The binding of NEDD8 to IAV PB2 attenuates protein stability and viral replication.^[^ ^14^ ^]^In addition to ubiquitination and ubiquitination‐like modifications, PARP12‐mediated modification of ADP ribosylation leads to the degradation of NS1 and NS3 of ZIKV, thereby inhibiting ZIKV replication.^[^ ^15^ ^]^

However, in some cases, the ubiquitination of viral proteins may also promote viral replication. For example, ubiquitination promotes the enzymatic activity of some viral proteins: Ebola Virus (EBOV) protein VP35 (an inhibitor of type I interferon);^[^ ^5^ ^]^ PB2 (polymerase basic protein 2),^[^ ^16^ ^]^NP nucleoprotein of IAV (promotes viral replication and assembly);^[^ ^17^ ^]^ HCV NS2 (promotes viral assembly),^[^ ^18^ ^]^thereby enhancing viral replication. Recently, Giraldo et al. reported that the E protein of ZIKV undergoes K63‐type ubiquitination by TRIM7, promoting viral binding to its receptor and contributing to viral invasion.^[^ ^19^ ^]^ Subsequently, LAMR1 and USP38 were reported to remove the ubiquitination of the E protein and inhibit the above process.^[^ ^20^ ^]^ It is also attractive to give examples of recent studies regarding new PTMs, such as succinylation and lactylation. Lactylation induced by porcine reproductive and respiratory syndrome virus (PRRSV) activates the expression of heat shock 70 kDa protein 6 (HSPA6), which promotes viral growth by impairing IFN‐β induction.^[^ ^21^ ^]^Another example is the dual modification of mitochondrial antiviral signaling protein (MAVS) at lysine 7, involving sirtuin3 (SIRT3)‐catalyzed deacetylation and sirtuin5 (SIRT5)‐catalyzed desuccinylation, which orchestrates antiviral innate immunity.^[^ ^22^ ^]^

For arboviruses, the studies of virus acquisition, replication, and transmission in arthropod vectors are also important. As evolutionarily conserved biological processes, protein translational modification systems also play an important regulatory role in mosquito vectors. For example, Gestuveo et al. systematically investigated ZIKV C protein interactions with proteins in mosquito cells and found that ubiquitination is essential for viral deconjugation of the capsid. Ubiquitination of C protein is found to be conserved in both animal host and vector cells.^[^ ^23^ ^]^This study suggests that PTMs to viral proteins exert conserved biological functions in both the host and the vector, critical to the life history of a multi‐host virus such as an arthropod‐borne virus. Another study by Paradkar et al. found that Cul4A/B, the E3 ligase of *Culex* mosquitoes, is essential for degrading mosquito STAT (Signal Transducer and Activator of Transcription) and thus promotes WNV infection of mosquitoes.^[^ ^24^ ^]^In contrast, a study in *Aedes aegypti* showed that the production of DENV infectious viral particles depends on mosquitoes' ubiquitin‐proteasome system. RNAi interfered with the ubiquitin‐proteasome system of mosquitoes and significantly reduced the release of infectious viral particles.^[^ ^25^ ^]^On the other hand, Troupin et al. found a Ub3881 ubiquitin gene in mosquitoes may degrade DENV E protein and inhibit viral replication.^[^ ^26^ ^]^Stokes et al. found that the ubiquitin‐like SUMOylation exerts an inhibitory effect on arbovirus infection of mosquitoes and that knockdown of the core genes of mosquito SUMOylation (SUMO, Ubc9, and PIAS) resulted in elevated levels of arbovirus replication.^[^ ^27^ ^]^These studies have demonstrated that ubiquitination and ubiquitination‐like modifications regulate arbovirus infection in mosquito vectors. However, how the mosquito ubiquitination system acts on specific viral proteins and performs roles that are beneficial (Pro‐viral) or detrimental (Anti‐viral) to viral replication remains largely unknown.

The NS1 protein of arboviruses is not only involved in viral replication and regulation of host immunity, but also essential for viral infection of mosquito vectors.^[^ ^28^ ^]^Although some studies have shown that certain mammalian E3 ligases can ubiquitinate flavivirus NS1 protein, the specific sites of action and types of modifications have not been identified.^[^ ^29^ ^]^The significance of these modifications in viral evolution and expansion has also not been elucidated.

To systematically investigate the role of protein PTMs in arbovirus infection of hosts and vectors, we employed a high‐resolution protein profiling platform to study the PTMs that occur in viral proteins during ZIKV infection. We observed that the E3 ubiquitin ligase WWP2 mediates the ubiquitination of NS1 in mammalian hosts. Interestingly, a protein homologous to WWP2, Su(dx), is present in mosquito vectors and can ubiquitinate NS1 as well. The ubiquitination of NS1 then plays different roles in the host and vector. The present study provides new insights into our understanding of the adaptive evolution of ZIKV in hosts and vectors.

## 2 Results

### 2.1 Post‐Translational Modifications of ZIKV Proteins

**Figure 1:**
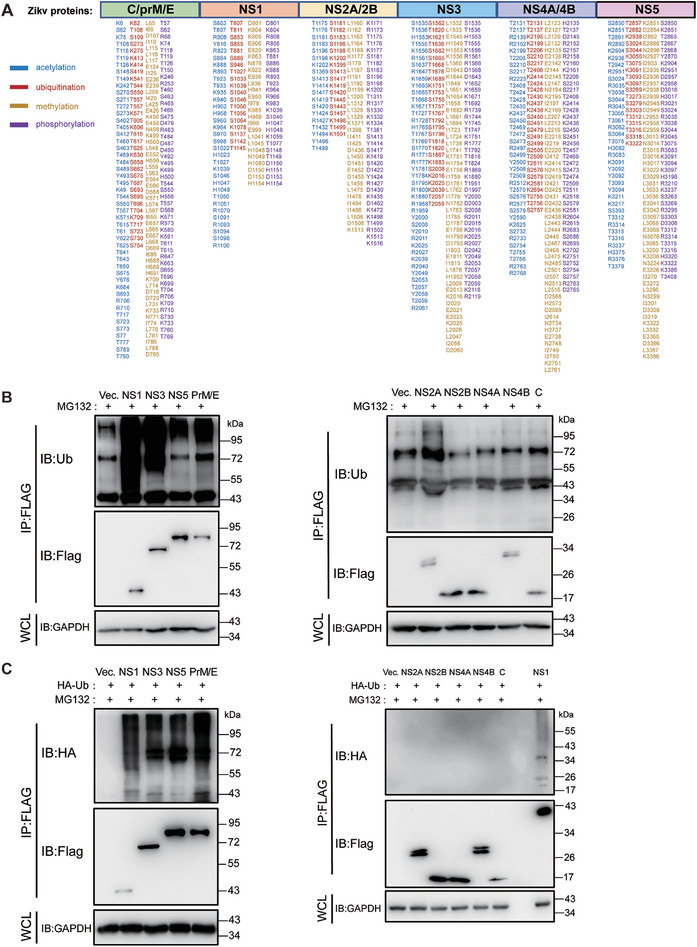
Post‐translational modification of ZIKV viral proteins. A) SH‐sy5y cells were infected with ZIKV (MR766) at a multiplicity of infection (MOI) of 1, and cell samples were collected 48 h post‐infection. Mass spectrometry was employed to analyze the acetylation, methylation, phosphorylation, and ubiquitination patterns of viral proteins. B and C) 293T cells were transfected with plasmids encoding various ZIKV proteins tagged with Flag (NS1‐Flag, NS3‐Flag, NS5‐Flag, PrM/E‐Flag, NS2A‐Flag, NS2B‐Flag, NS4A‐Flag, NS4B‐Flag, or C‐Flag). B) In another set of experiments, these proteins were co‐transfected with a plasmid expressing ubiquitin tagged with HA (Ub‐HA). C) After 24 h, cells were treated with MG132 (5 µM) for 4 h. Cell lysates were collected, and immunoprecipitation with Flag antibody‐coupled magnetic beads was performed. The ubiquitination levels of viral proteins were then analyzed by Western Blot. The presented data are representative of three independent experiments.

To investigate the significance of PTMs on viral proteins in flavivirus infection, we infected human neuroblastoma cells (SH‐sy5y) with the ZIKV MR766 strain. Total cellular proteins were analyzed using LC‐MS/MS, and the amino acid sequences of the MR766 strain were queried using Proteome Discoverer software as a database. ZIKV‐encoded genome proteins (C, PrM, E, NS1, NS2A, NS2B, NS3, NS4A, NS4B, and NS5) were identified with sequence coverage ranging from 60% to 81%. Mass spectrometry data analyzed using Proteome Discoverer 2.2 software indicated that potential acetylation, methylation, phosphorylation, and ubiquitination (or ubiquitination‐like) modifications were present in ZIKV proteins (**Figure** **1**A). Most viral proteins underwent multiple modifications at different amino acid residues, and the same amino acid residue was also found to undergo various modifications (Figure 1A). Notably, we observed that several viral proteins could undergo ubiquitination at multiple amino acid residues, including lysine, serine, and threonine residues.

To validate the mass spectrometry results mentioned above, we examined three structural and seven nonstructural proteins of ZIKV for the presence of ubiquitination in cells. ZIKV proteins were overexpressed separately in 293T cells, and the cells were treated with MG132 24 h after transfection. The ubiquitination level of viral proteins was detected using a Ub antibody after immunoprecipitation of ZIKV proteins. The results revealed significant ubiquitination in NS1, NS3, NS5, and PrM/E proteins, whereas the ubiquitination in NS2A, NS2B, NS4A, NS4B, and C proteins was not significant (Figure 1B). To further demonstrate the ubiquitination of the aforementioned ZIKV proteins, we co‐expressed the ZIKV proteins with HA‐tagged Ub and detected the ubiquitination level of the viral proteins with an HA antibody. The results agreed with Figure 1B, and the ubiquitination level of NS1, 3, and 5 was even more significant (Figure 1C).

### 2.2 E3 Ubiquitin Ligase WWP2 Interacts with ZIKV NS1

**Figure 2:**
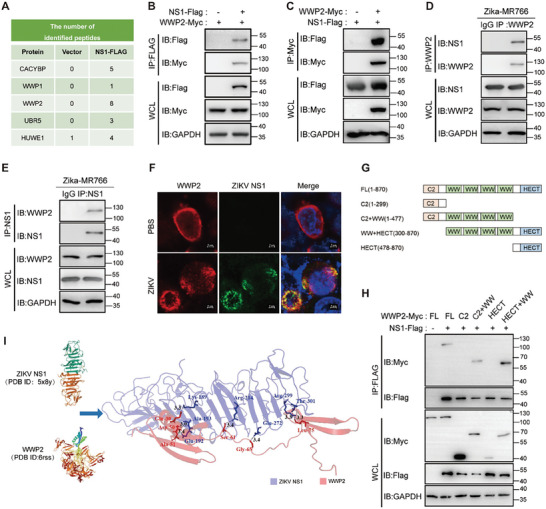
E3 ubiquitin ligase WWP2 interacts with NS1. A) NS1‐Flag was transfected into 293T cells, followed by ZIKV infection (MOI = 0.5) 24 h later. Immunoprecipitation of NS1‐Flag was performed with Flag antibody‐coupled magnetic beads 24 h post‐infection to analyze the ubiquitin‐associated enzymes interacting with NS1‐Flag protein using mass spectrometry. B–E) WWP2‐Myc was co‐transfected with NS1‐Flag expression plasmid in 293T cells. After 24 h, immunoprecipitation of NS1‐Flag was performed, and WWP2‐Myc protein was detected by Western Blot. B) In another set of experiments, immunoprecipitation of WWP2‐Myc was carried out, and NS1‐Flag protein was detected by Western Blot. C) In addition, 293T cells were infected with ZIKV (MOI = 1), and after 48 h, endogenous WWP2 was immunoprecipitated. ZIKV NS1 protein was then detected by Western Blot D), and endogenous WWP2 protein was detected by immunoprecipitation of ZIKV NS1 and Western Blot E). F) 293T cells were infected with ZIKV(MOI = 0.5) for 48 h. The intracellular localization of the endogenous WWP2 and NS1 proteins was observed using laser confocal imaging. G and H) Each of the WWP2 truncates was co‐transfected with NS1‐Flag in 293T cells, and the cells were collected after 24 h. Immunoprecipitation with NS1‐Flag was performed, and Western Blot detected the expression of Myc‐tagged truncated proteins. I) Molecular docking prediction results for WWP2‐WW and ZIKV NS1 proteins. The data presented are representative of three independent experiments.

Given that the NS1 protein is involved in many steps of the flavivirus life cycle, including viral replication, immune evasion, and pathogenesis,^[^ ^28^, ^30^ ^]^we searched for ubiquitin‐associated proteins that mediate the ubiquitination of the NS1 protein. Using IP‐MS analysis, we identified four E3 ubiquitin ligases (WWP1, WWP2, UBR5, and HUWE1) and one ubiquitin E3‐associated protein CACYBP (which can bind to E3 ligases and regulate ubiquitination of proteins),^[^ ^31^ ^]^ as potential interacting proteins of NS1 (**Figure** **2**A). To validate the interaction of the ubiquitin‐associated enzymes with NS1, we conducted co‐immunoprecipitation validation. The experimental results demonstrated that NS1 directly interacted with CACYBP (Figure S1A, Supporting Information), WWP1 (Figure S1A, Supporting Information), and WWP2 (Figure 2B,C). As WWP2 exhibited the highest number of peptides interacting with NS1 in the mass spectrometry results (Figure 2A), we further confirmed the interaction between NS1 and WWP2. Bidirectional immunoprecipitation experiments revealed that endogenously expressed WWP2 also interacts with NS1 in ZIKV‐infected cells (Figure 2D and E; Figure S2A, Supporting Information). Confocal microscopy experiments demonstrated the co‐localization of WWP2 with NS1 in the cells (Figure 2F; Figure S2B, Supporting Information). Additionally, GST pull‐down assays confirmed that WWP2 can directly interact with NS1 (Figure S2C, Supporting Information).

WWP2 comprises three functional structural domains: a C‐terminal HECT structural domain, four WW repeat structural domains, and an N‐terminal C2 structural domain. To find the specific domains where WWP2 interacts with NS1, we constructed four truncations of WWP2: C2 (1–299 aa), C2+WW (1–477 aa), WW+HECT (300–870 aa), and HECT (478–870 aa) (Figure 2G). Immunoprecipitation results demonstrated that the C2+WW and WW+HECT truncations interacted with NS1, whereas the C2 and HECT truncations did not display any affinity for NS1 (Figure 2H). These findings strongly suggest that the four WW structural domains of WWP2 play a pivotal role in mediating its interaction with NS1.To validate this assertion, we accessed the crystal structures of WWP2 and NS1 and conducted molecular docking. The outcomes corroborated that the WW structural domain of WWP2 interacts with NS1, with potential binding sites identified as Glu‐30, Asp‐50, Ala‐51, Ser‐61, Gly‐65, and Leu‐75 of WWP2‐WW, corresponding to ZIKV NS1 Lys‐189, Ala‐193, Glu‐192, Arg‐214, Glu‐272, and Thr‐301, respectively (Figure 2I).

### 2.3 WWP2 is a Key E3 Ligase for Ubiquitination and Degradation of NS1

**Figure 3:**
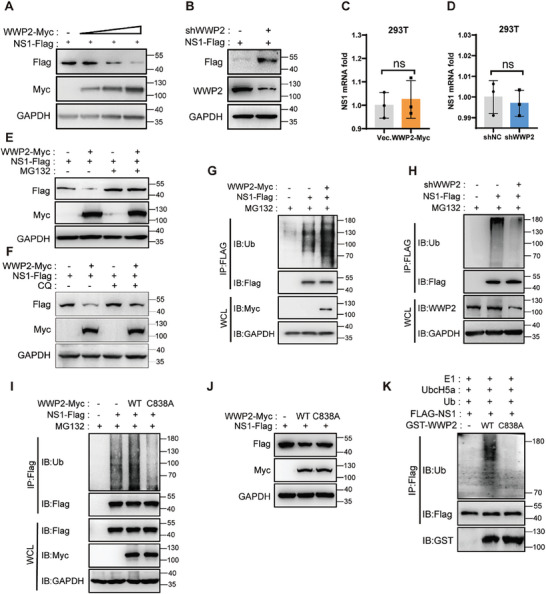
WWP2 ubiquitinates NS1 and leads to NS1 degradation. A and B) NS1‐Flag and WWP2‐Myc/shWWP2 (1 µg) were co‐transfected in 293T cells, and the protein levels of NS1‐Flag were detected by Western Blot 48 h later. C and D) NS1‐Flag and WWP2‐Myc/shWWP2 (1 µg) were co‐transfected in 293T cells, and the mRNA levels of NS1 were detected by qRT‐PCR 48 h later. E) NS1‐Flag and WWP2‐Myc were co‐transfected in 293T cells, which were then treated with MG132 (5 µM, 4 h) 24 h later. The protein levels of NS1‐Flag were detected by the Western Blot method. F) NS1‐Flag and WWP2‐Myc were co‐transfected in 293T cells. 24 h later, cells were treated with Chloroquine (10 µM, 6 h). The protein levels of NS1‐Flag were detected by the Western Blot. G and H) NS1‐Flag and shWWP2/WWP2‐Myc (1 µg) were co‐transfected in 293T cells and treated with MG132 (5 µM) for 4 h after 48 h. The ubiquitination level of NS1‐Flag protein was detected by the Western Blot method after immunoprecipitation of NS1‐Flag. I and J) NS1‐Flag and WWP2‐Myc (WT or C838A) plasmids were co‐transfected in 293T cells. After 24 h, immunoprecipitation of NS1‐Flag was performed, and the ubiquitination levels of NS1‐Flag protein were detected by Western Blot (F). The protein levels of NS1‐Flag were detected by Western Blot (G). K) NS1‐Flag, purified WWP2 (or WWP2‐C838A), E1 (Hdm2), and E2 (UbcH5a) were incubated for 1 h in the presence of ATP. The in vitro ubiquitination level of NS1 was analyzed by Western Blot. The data presented are representative of three independent experiments. ns, non‐significant (Student's t‐test).

To investigate the potential regulatory impact of the five ubiquitin‐associated enzymes on NS1 protein expression, we co‐expressed NS1 along with varying concentrations of CACYBP, WWP2, HUWE1‐HECT (the HECT structural domain of HUWE1), UBR5, or WWP1 in 293T cells (**Figure** **3**A; Figure S1B, Supporting Information). Subsequently, we assessed the NS1 protein levels 24 h post‐transfection using the Western Blot. The results revealed that CACYBP, WWP2, and HUWE1‐HECT exhibited a dose‐dependent down‐regulation effect on NS1 protein levels (Figure 3A; Figure S1B, Supporting Information). Conversely, UBR5 and WWP1 did not exert a significant influence on NS1 protein levels (Figure S1B, Supporting Information). Additionally, the knockdown of endogenous CACYBP (Figure S1C, Supporting Information) and WWP2 (Figure 3B) resulted in a noteworthy up‐regulation of NS1 protein levels. Furthermore, neither overexpression nor knockdown of WWP2 affected NS1 mRNA expression (Figure 3C,D).

The principal protein degradation pathways encompass the lysosomal and proteasomal pathways. To discern through which pathway CACYBP or WWP2 induces the degradation of NS1, we co‐expressed NS1 with CACYBP‐Myc (Figure S1D, Supporting Information) or WWP2‐Myc (Figure 3E) in 293T cells. Subsequently, we treated the cells with the proteasome inhibitor MG132 (5 µM) and lysosomal autophagy inhibitor CQ (50 µM). Western blot results demonstrated that the down‐regulatory effects of CACYBP and WWP2 on NS1 protein were abrogated upon MG132 treatment (Figure 3E; Figure S1D, Supporting Information). Additionally, WWP2‐mediated degradation of NS1 protein is not dependent on lysosomes (Figure 3F). This observation implies that the regulatory effects of CACYBP and WWP2 on NS1 protein degradation are mediated through the proteasome pathway.

Subsequently, we investigated the impact of CACYBP and WWP2 on the ubiquitination level of NS1 proteins by co‐expressing NS1‐Flag with CACYBP‐Myc or WWP2‐Myc in 293T cells. The results demonstrated that the overexpression of CACYBP did not exert a significant effect on the ubiquitination of NS1, whereas the overexpression of WWP2 notably enhanced the ubiquitination of NS1(Figure 3G; Figure S1E, Supporting Information). To further elucidate the role of endogenous CACYBP and WWP2 in NS1 ubiquitination, we assessed NS1 ubiquitination levels following the knockdown of CACYBP or WWP2 in cells. The findings indicated that the knockdown of CACYBP did not significantly influence the ubiquitination level of NS1, while the knockdown of WWP2 resulted in a reduction in NS1 ubiquitination (Figure 3H; Figure S1F, Supporting Information). These results imply that WWP2 functions by ubiquitinating NS1 protein, thereby instigating a proteasome pathway‐dependent degradation process.

The aforementioned findings underscore WWP2, an E3 ubiquitin ligase, as a pivotal enzyme facilitating the ubiquitination and proteasome‐dependent degradation of NS1 proteins. Subsequently, we investigated whether the regulatory influence of WWP2 on NS1 proteins is contingent upon its E3 ubiquitin ligase activity. The cysteine residue (Cys) at position 838 of WWP2 has been identified as its ubiquitin ligase active site.^[^ ^32^ ^]^Our results demonstrated that the mutation of cysteine at position 838 of WWP2 abrogated its ability to ubiquitinate NS1 (Figure 3I), consequently impeding the subsequent degradation of NS1 (Figure 3J). Moreover, in vitro ubiquitination experiments revealed that the WT WWP2, but not its enzyme‐inactive mutant WWP2 (C838A), could directly target NS1 for ubiquitination (Figure 3K). These findings strongly suggest that the regulation of NS1 protein by WWP2 is contingent upon its E3 ubiquitin ligase activity.

### 2.4 WWP2 is Upregulated During ZIKV Infection

**Figure 4:**
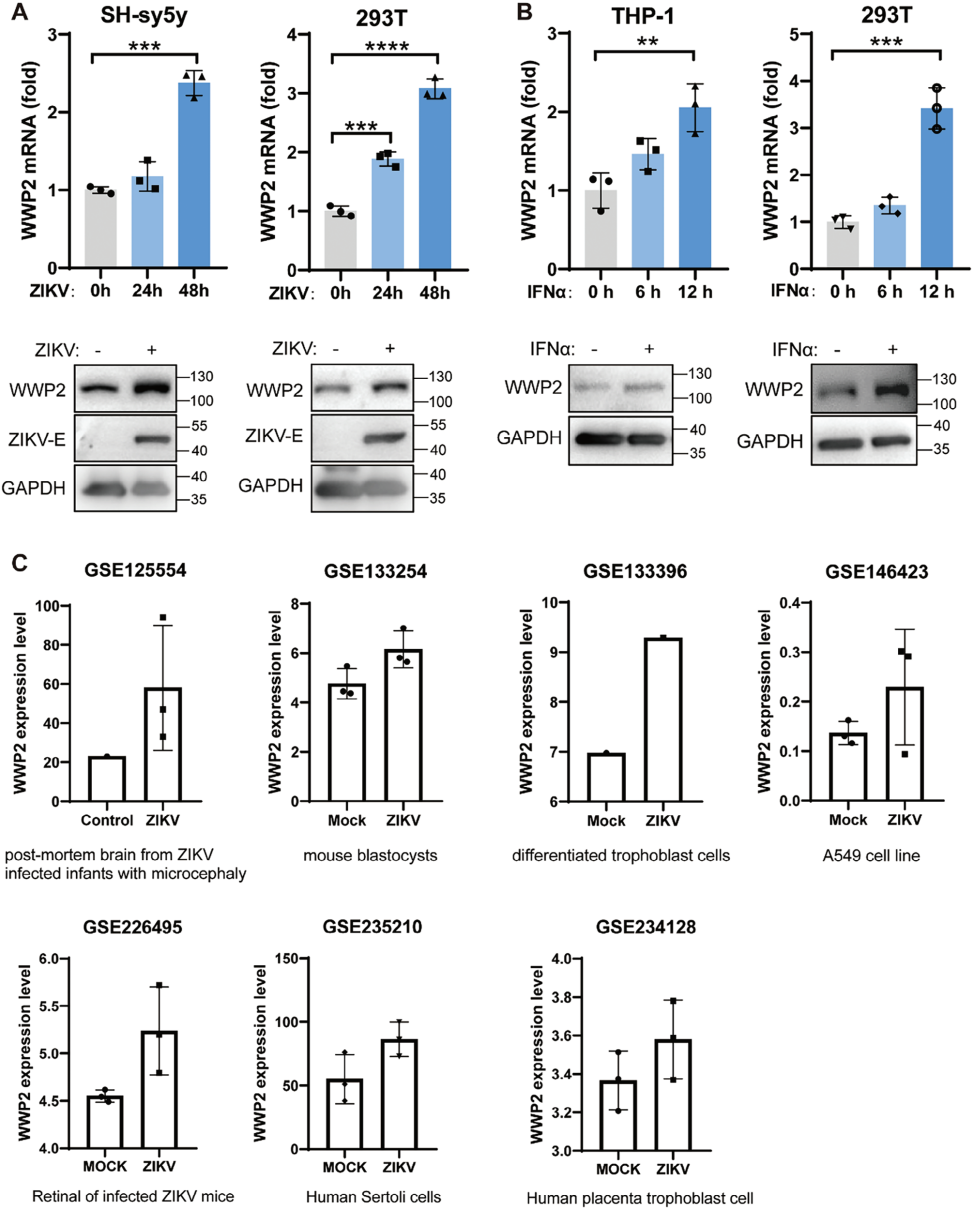
WWP2 expression was upregulated during ZIKV infection. A) SH‐sy5y or 293T cells were infected with ZIKV (MR766) (MOI = 0.5). WWP2 levels was analyzed by qRT‐PCR and Western Blot. B) THP‐1 or 293T cells were treated with IFN‐α (500 U ml^−1^) for 6 h, and WWP2 levels were determined using qRT‐PCR and Western Blot. C) WWP2 expression was up‐regulated during ZIKV infection based on GEO databases. Data are representative of 3 independent experiments and presented as mean ± SD. ** *P* < 0.01, and *** *P* < 0.001, **** *P* < 0.0001 (Student's t‐test).

We assessed the impact of ZIKV infection on WWP2 levels in SH‐sy5y and 293T cells using qRT‐PCR and Western Blot. The results unveiled an up‐regulation of WWP2 expression during ZIKV infection (**Figure** **4**A). Intriguingly, WWP2 was considered as an ISG according to the Interferome database (http://interferome.org/). In line with this, WWP2 expression increased following interferon‐alpha (IFNα) stimulation (Figure 4B).

To gain further insights, we delved into transcriptome sequencing data from in vivo and in vitro ZIKV infection models available in the GEO database. Among these datasets, GSE125554 utilized a systems biology approach, integrating transcriptomic data from postmortem brains of newborns with congenital Zika syndrome (CZS); GSE133254 provided transcriptomic data from mouse blastocysts infected with ZIKV; GSE133396 presented transcriptomic data from human trophoblast cells infected with ZIKV; GSE146423 included transcriptomic data from the human lung cancer cell line A549 infected with ZIKV; GSE226495 provided mRNA expression profiles in the retina of ZIKV‐infected mice; GSE235210 presented genome‐wide RNA‐seq to human Sertoli cells infected with ZIKV; and GSE234128 identified the differentially expressed genes through transcriptome analyses ZIKV‐infected human placenta trophoblast cells. Across these diverse models, the expression level of the WWP2 gene consistently exhibited an up‐regulation following ZIKV infection (Figure 4C). These collective findings imply a significant role for host WWP2 in the process of ZIKV infection.

### 2.5 WWP2 Suppresses ZIKV Infection

**Figure 5:**
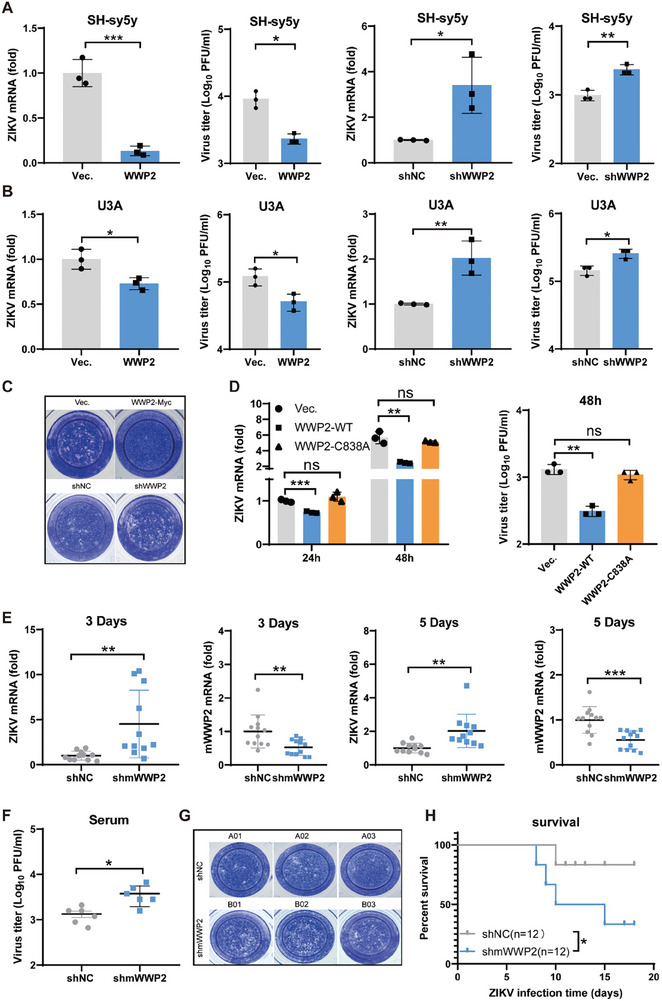
WWP2 restricts ZIKV infection. A–C) SH‐sy5y (A)/U3A (B) cells were infected with lentivirus overexpressing or knocking down WWP2 (MOI = 10). Subsequently, cells were infected with ZIKV (MOI = 0.5) 48 h later. Viral mRNA levels in the cells were detected 24 h later using qRT‐PCR. Viral load in the supernatant was visualized by TCID50, and the infectious viral load in U3A supernatants was determined by plaque assay (C). D) WWP2‐Myc (WT or C838A) plasmid was transfected into 293T cells and infected with ZIKV (MOI = 0.5) after 24 h. Cellular RNA was extracted at 24 and 48 h, and the viral RNA levels were analyzed by qRT‐PCR. The viral supernatant titer after 48 h was determined by TCID50. E‐H) *Ifnar1^−/−^* mice (6 weeks old, 12 mice per group) were injected with 5 × 10^7^ PFU shmWWP2 lentivirus via the tail‐vein route; 7 days later, mice were injected intraperitoneally with 10^7^ PFU ZIKV. Hemocytes and serum were collected on days 3 and 5. Blood cell RNA was extracted, and qRT‐PCR was used to detect the RNA content of ZIKV and shmWWP2 in the cells E). Viral titers in the serum of mice on day 5 were detected by TCID50 F). Infectious virus in the serum of mice on day 5 was detected by the plaque assay G). The status and survival of mice were recorded by daily observation (* *P* < 0.05, Log‐rank test) (H). Data are representative of 3 independent experiments and presented as mean ± SD. ns, non‐significant, * *P* < 0.05, ** *P* < 0.01, and *** *P* < 0.001 (Student's t‐test).

To elucidate the impact of WWP2 on ZIKV infection, we employed overexpression of WWP2 in diverse cell types and evaluated ZIKV infectivity by measuring both the viral RNA level within cells and viral particle abundance in cell supernatants. The results unequivocally demonstrated a significant reduction in virus levels in both cells and supernatants upon WWP2 overexpression compared to the control. Conversely, viral infection exhibited an increase following the knockdown of intracellular WWP2 expression (**Figure** **5**A; Figure S3A,B, Supporting Information). NSC2805, a natural chemical inhibitor of WWP2, was employed to investigate the role of WWP2 in ZIKV infection in host cells. The results demonstrated that NSC2805 promoted viral replication in 293T cells (Figure S3C, Supporting Information). These findings strongly indicate that WWP2 exerts an inhibitory effect on ZIKV infection within cells.

Interferon serves as the primary line of defense against viral invasion, and previous reports have suggested that WWP2 may negatively regulate the TLR3 signaling pathway, impacting interferon production.^[^ ^33^ ^]^To ascertain whether WWP2 still inhibits ZIKV replication in the absence of the interferon system, we examined the effect of WWP2 on ZIKV replication in Vero E6 cells, which are defective in the IFN pathway, and U3A cells, which lack STAT1. The results revealed a significant decrease in viral infection after WWP2 overexpression compared with controls (Figure 5B; Figure S3D, Supporting Information) and an increase in viral replication after the knockdown of intracellular WWP2 expression (Figure 5B). Plaque assays further demonstrated a significant reduction in the viral titer following WWP2 overexpression, while it increased after WWP2 knockdown (Figure 5C). These findings collectively indicate that the regulatory effect of WWP2 on ZIKV is not contingent upon its influence on the IFN pathway.

To scrutinize the necessity of E3 ubiquitin ligase activity for WWP2 to exert an antiviral effect, we overexpressed WWP2 WT or C838A enzyme‐inactive mutants in 293T cells and analyzed the levels of viral RNA replication and the virus titer in the cellular supernatants after infecting the cells with ZIKV (Figure 5D). The results revealed that WWP2 enzyme‐inactive mutants lost the ability to inhibit ZIKV replication, which strongly suggests that the ability of WWP2 to regulate ZIKV replication is contingent upon its E3 ubiquitin ligase activity. Furthermore, we detected the viral load after treating the cells with MG132. The results showed that WWP2 lost its inhibitory effect on ZIKV infection after MG132 treatment (Figure S3E, Supporting Information). This reinforces the idea that WWP2 inhibits ZIKV infection by regulating the NS1 protein, thereby preventing ZIKV infection.

To further substantiate the inhibitory effect of WWP2 on ZIKV replication, we administered shmWWP2 lentivirus into *Ifnar1*‐deficient C57BL/6 mice^[^ ^34^ ^]^via the tail‐vein route. Seven days later, mice were intraperitoneally injected with ZIKV MR766 virus. The knockdown efficiencies of mWWP2 and the RNA content of ZIKV in murine blood cells on days 3 and 5 were assessed by qRT‐PCR, respectively (Figure 5E). Additionally, the viral titers in the sera of the mice on the fifth day were determined using TCID_50_ (Figure 5F) and plaque assays (Figure 5G). The results indicated a significant increase in viral load in vivo after mWWP2 was knocked down compared to WT mice. Furthermore, the mortality rate after ZIKV infection dramatically increased in mWWP2 knockdown mice (Figure 5H). These in vivo experiments further underscore the pivotal role of WWP2 in inhibiting ZIKV infection. Additionally, we infected WT and WWP2‐deficient (*Wwp2* ^−/−^)^[^ ^35^ ^]^ suckling mice via transcranial cerebral injection of ZIKV. *Wwp2* ^−/−^ mice showed higher viral load, more severe tissue damage, and higher mortality rate (Figure S3F–H, Supporting Information). Furthermore, we employed WT and *Wwp2* ^−/−^ pregnant mice and injected ZIKV into their tail veins. We also observed a higher viral load in the blood of *Wwp2* ^−/−^ pregnant mice compared to WT controls (Figure S3I, Supporting Information). The results indicate that WWP2 reduces the pathogenesis of ZIKV in mice.

### 2.6 WWP2 Ubiquitinated Amino Acids K265 and K284 of ZIKV NS1

**Figure 6:**
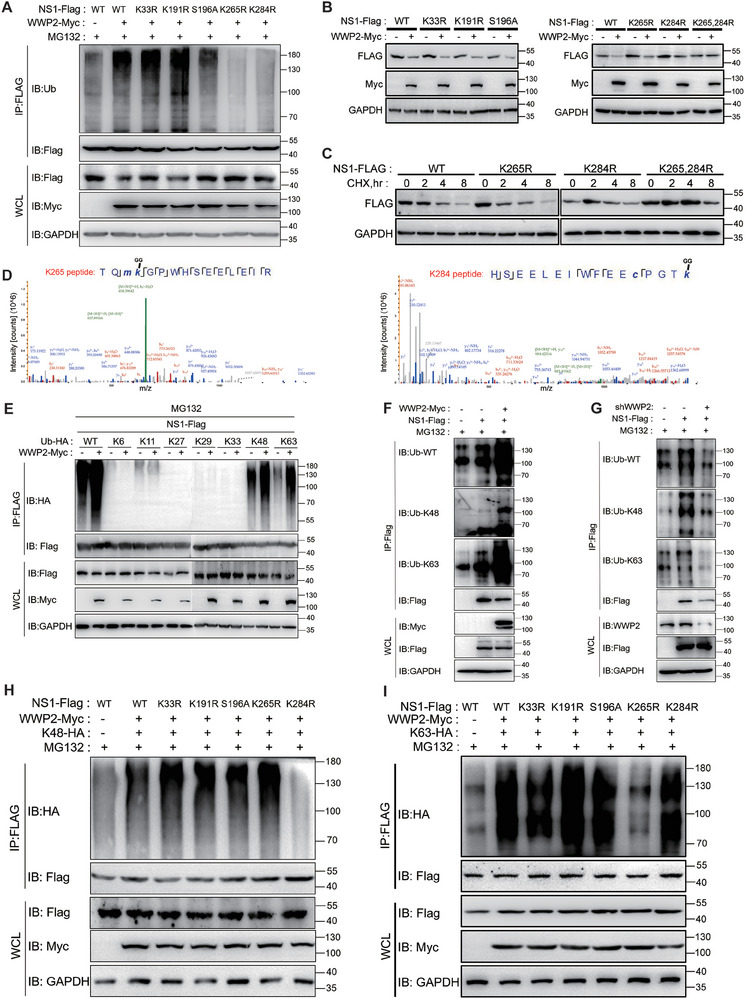
WWP2 ubiquitinates amino acids K265 and K284 of ZIKV NS1. A and B) WWP2‐Myc was co‐transfected with NS1‐WT or its mutants in 293T cells for 24 h. After 24 h, the cells were treated with MG132 (5 µM) for 4 h. NS1‐Flag was immunoprecipitated, and the ubiquitylation level of NS1 proteins was detected by Western Blot A). The protein level of NS1 was detected by Western Blot B). C) NS1‐WT or its mutants were transfected into 293T cells and treated with actinomycin ketone CHX (50 µM) for 0, 2, 4, and 8 h after 24 h. NS1 protein levels were detected by Western Blot. D) Secondary mass spectrometry analysis of ubiquitinations at positions K265 and K284 of NS1. E) WWP2‐Myc, NS1‐Flag, and ubiquitin molecule mutant plasmids (K6, K11, K27, K29, K33, K48, and K63) were co‐transfected into 293T cells. After 24 h, the cells were treated with MG132 (5 µM, 4 h), and NS1‐Flag was immunoprecipitated. NS1 proteins were detected by Western Blot method, and the ubiquitination level was assessed. F and G) WWP2‐Myc or shWWP2 (1 µg), NS1‐Flag, were co‐transfected into 293T cells. After 24 h, the cells were treated with MG132 (5 µM, 4 h), and NS1‐Flag was immunoprecipitated. NS1 proteins were detected by Western Blot, and the ubiquitination types of NS1 were detected using K48 and K63 antibodies. H and I) WWP2‐Myc, NS1‐Flag individual point mutants, and ubiquitin molecule mutant K48/K63‐HA were co‐transfected into 293T cells. After 24 h, the cells were treated with MG132 (5 µM, 4 h), and immunoprecipitated with NS1‐Flag. The level of ubiquitination of NS1 proteins was detected by Western Blot. Data are representative of 3 independent experiments.

To elucidate the specific sites where WWP2 catalyzes ubiquitination on the NS1 protein, we employed the CKSAAP bioinformatics website to predict potential ubiquitination sites. Several loci with high reproducibility and confidence—K33, K191, S196, K265, and K284—were selected from the analyzed results. Single‐point mutants were constructed for these five amino acids. WWP2‐Myc was co‐transfected with NS1‐WT or its mutants in 293T cells, followed by treatment with MG132. After immunoprecipitation using Flag antibody‐coupled magnetic beads, the ubiquitination level of NS1 proteins (**Figure** **6**A) and the protein expression level of NS1 were assessed (Figure 6B). The results revealed that WWP2 no longer regulated the ubiquitination and protein level of NS1 after mutation of both Lys 265 and Lys 284 locus (Figure 6A,B).

To further investigate the impact of these mutations on NS1 protein stability, we transfected NS1‐WT or its mutants into 293T cells. We detected NS1 protein levels after treatment with the protein synthesis inhibitor actinomycin ketone CHX (50 µM) for 0, 2, 4, and 8 h (Figure 6C). The experimental results demonstrated that the mutation of a single site, K265 or K284, did not significantly affect the degradation rate of the NS1 protein. In contrast, simultaneous mutation of both sites significantly enhanced the stability of the NS1 protein. This suggests that ubiquitination at the K265 and K284 sites plays a crucial role in affecting the stability of the NS1 protein. Finally, secondary mass spectrometry further corroborated the presence of ubiquitinations at the K265 and K284 sites of NS1 (Figure 6D). These collective results indicate that K265 and K284 of NS1 are ubiquitination sites catalyzed by WWP2.

To delve deeper into the specific types of ubiquitination that WWP2 regulates on NS1, we conducted an analysis using ubiquitin molecule mutants (K6, K11, K27, K29, K33, K48, and K63, wherein the K of all other sites is mutated to R). The experimental results revealed that the overexpression of WWP2 significantly promoted K48 and K63 types of ubiquitination, while it did not impact other ubiquitination types (Figure 6E). We further confirmed the ubiquitination types of NS1 in human cells using K48 and K63 antibodies, which clarified that WWP2 mediates K48 and K63 ubiquitination of NS1 (Figure 6F,G).

To pinpoint the specific site on the NS1 protein where K48 and K63 type ubiquitination occurs, we co‐transfected WWP2‐Myc, NS1‐Flag point mutants (K33R, K191R, S196A, K265R, and K284R), and ubiquitin molecule mutants (K48/K63‐HA) in 293T cells. After 24 h, the cells were treated with MG132, and the ubiquitination level of NS1 proteins was assessed following immunoprecipitation with Flag antibodies. The experimental results elucidated that WWP2 catalyzed the K48 type ubiquitination of K284 (Figure 6H) and the K63 type ubiquitination of K 265 of the NS1 protein (Figure 6I).

**Figure 7:**
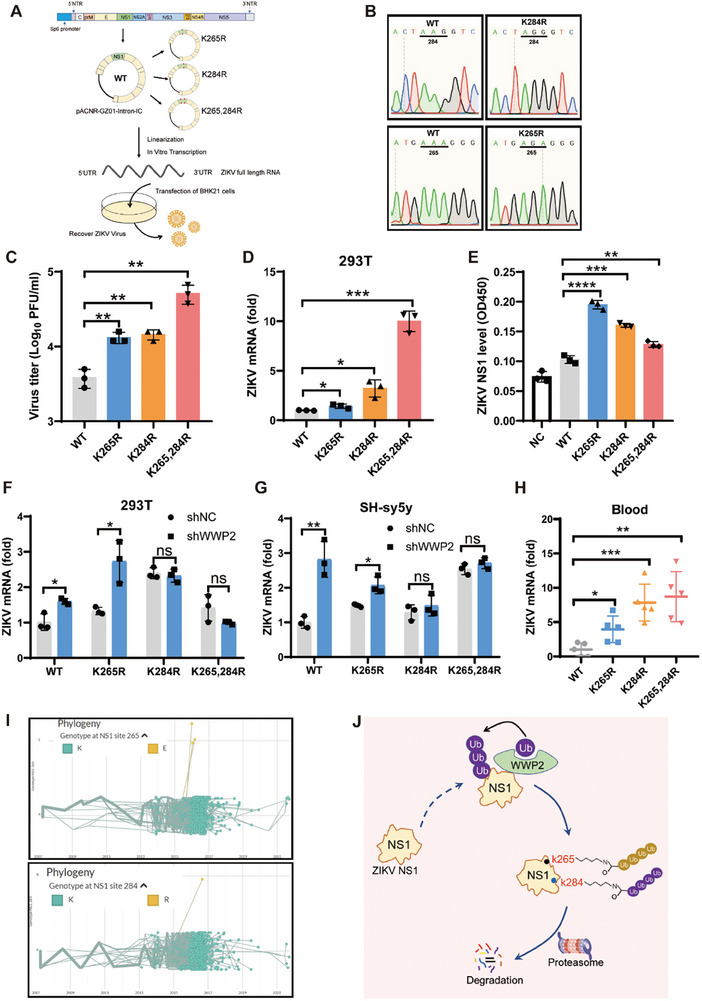
Amino acid mutation at position NS1 K265, K284 alters ZIKV virulence. A and B) Schematic diagram of the ZIKV packaging process (A): The full‐length plasmid of the 2016 GZ01 strain was used as a template. The full‐length plasmid of the NS1 point‐mutated K265R, K284R, and K265/284R viral genomes was obtained by targeted mutagenesis. The full‐length plasmid was transfected with RNA into BHK21 cells after in vitro transcription, and the viral supernatant was collected after culture to obtain the WT viruses and mutant viruses (B). C–E) WT, K265R, K284R, and K265/284R viruses were packaged with the same mass of RNA, and the titer of the viral particles was detected by TCID50 (C). 293T cells were infected with the same titer of the mutant viruses (MOI = 1), and the intracellular viral load was detected by qRT‐PCR 48 h later (D). The same titer of mutant viruses was used to infect 293T cells (MOI = 1), and the level of ZIKV NS1 in the supernatant was detected by ELISA after 72 h (E). F and G) 293T (F) and SH‐sy5y (G) cells were infected with lentiviruses knocking down the expression of WWP2, and then infected with WT and mutant viruses after 48 h. Cells were collected after 48 h to extract the RNA, and the viral load in the cells was detected by qRT‐PCR. H) *Ifnar1^−/−^* mice were infected with 10^7^ PFU viruses (WT, K265R, K284R, and K265/284R), and viral loads were detected by qRT‐PCR on day 5 after infection. I) A search of the Virus Sequence Library (https://nextstrain.org) revealed the existence of a naturally occurring strain of ZIKV NS1 mutated at amino acid positions 265/284. J) Model for regulation of ZIKV NS1 by WWP2. Data are representative of 3 independent experiments and presented as mean ± SD. ns, non‐significant, * *P* < 0.05, ** *P* < 0.01, and *** *P* < 0.001, **** *P* < 0.0001 (Student's t‐test).

The preceding experimental results strongly indicate that WWP2 catalyzes the ubiquitination of the K265 and K284 sites of NS1, leading to its subsequent degradation via the proteasome pathway. To explore the effects of the K265 and K284 loci of NS1 on the infectivity of ZIKV, we generated infectious virus particles mutated at these two loci through a reverse genetic system. The full‐length plasmid pACNR‐GZ01‐Intron‐IC of the GZ01 strain,^[^ ^36^ ^]^prevalent in China in 2016, served as a template. Fixed‐point mutations were introduced to obtain NS1 K265R, K284R, and K265/284R viral genome mutants. Following SP6 promoter in vitro transcription, the RNA was transfected into BHK21 cells, and the viral supernatant was collected to obtain WT and mutant viruses (**Figure** **7**A,B). WT, K265R, K284R, and K265/284R mutant viruses were packaged with an equivalent amount of RNA. The titers of the viruses recovered by the reverse genetic system were measured by TCID_50_ assay with Vero E6 cells. The results showed that K265R and K284R mutant viruses were more virulent than WT viruses (Figure 7C). Infection of 293T cells with the same titer (MOI = 1) of WT and mutant viruses revealed that the K265R and K284R mutant viruses exhibited higher infectivity than the WT viruses, and the K265/K284R double mutant viruses displayed increased virulence in 293T cells (Figure 7D).

The NS1 protein is secreted into the supernatant by infected host cells and serves as a marker for infection diagnosis. Therefore, we also examined the impact of the K265R and K284R mutations on NS1 protein secretion. 293T cells were infected with the same titer of WT and mutant viruses, and ELISA was employed to measure the level of ZIKV NS1 in the supernatant after 72 h. The results indicated that the NS1 protein secretion of K265R, K284R, and K265/284R mutant viruses was higher than that of WT viruses (Figure 7E). These findings suggest that WWP2 inhibits ZIKV infection and the K265R, K284R mutations enhance ZIKV infection.

To assess whether this phenomenon is mediated through WWP2, the viral load in cells was initially examined by knocking down WWP2 and infecting the cells with WT and mutant viruses. The results demonstrated that after knocking down the expression of WWP2, there was no difference in the ability of K265/K284R mutant viruses to infect cells compared to WT viruses. This indicates that the K265R, K284R mutation attenuated the ability of WWP2 to regulate ZIKV in vitro. (Figure 7F,G). To assess the infectivity of K265R and K284R mutant viruses in vivo, we infected *Ifnar1* ^−/−^ mice with 10^7^ PFU of both WT and mutant viruses. After 5 days, we measured the viral load in the peripheral blood of the mice using RT‐qPCR. The results indicate that the K265R and K284R mutations in NS1 enhance the virus's ability to infect the host in vivo. (Figure 7H).

To investigate whether mutations at the NS1 protein K265 and K284 sites occur naturally, we searched the Viral Sequence Library (https://nextstrain.org) and revealed a K‐to‐E mutation at position 265 of NS1 in the 2016 Mexican, Brazilian, and Colombian ZIKV strains (Figure 7I), and a K‐to‐R mutation at position 284 in the 2016 Thai strain (Figure 7I). These evidence suggest the existence of viral strains with amino acid mutations at positions 265/284 of ZIKV NS1 in nature, leading to amino acid residues that are not conducive to ubiquitination.

Based on these data, we identified that WWP2 selectively targets the K265 and K284 sites of the ZIKV NS1 protein, promoting K63 and K48‐type ubiquitination, respectively. Consequently, this ubiquitination process triggers the degradation of NS1 via the proteasome pathway, ultimately inhibiting viral infection in mammalian hosts (Figure 7J).

### 2.7 WWP2 is a Restriction Factor for Several Flaviviruses

**Figure 8:**
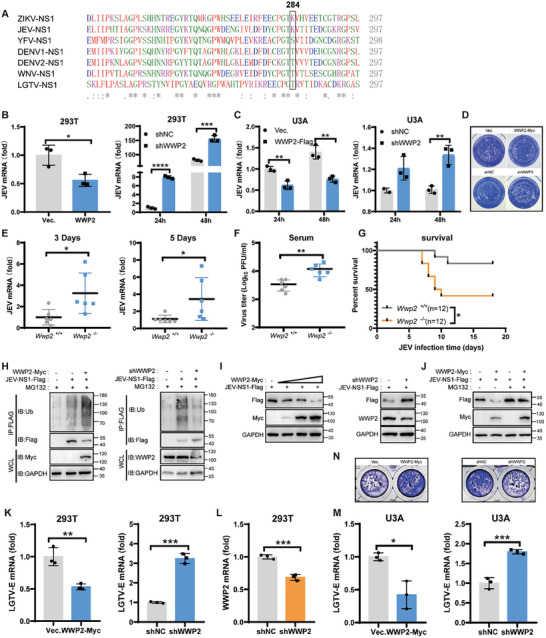
WWP2 is a broad‐spectrum arthropod‐borne flavivirus suppressor. A) Conservation of site 284 of the arthropod‐borne flavivirus NS1 protein. B–D) After overexpression or knockdown of WWP2 in 293T (B) and U3A (C) cells, the cells were infected with JEV (MOI = 0.5), and cellular RNA was extracted after 24 and 48 h. The viral content of the cells was detected by qRT‐PCR; the amount of infectious viruses in the supernatant of U3A was detected by plaque assay (D). E–G) Using WT and *Wwp 2^−/−^* mice, 10^7^ PFU JEV (SA14) was injected intraperitoneally, and hemocytes and serum were collected by orbital blood sampling on days 3 and 5, respectively. Blood cell RNA was extracted, and the amount of JEV in the cells was detected using qRT‐PCR (E); the viral titer in the serum of mice on day 5 was detected by TCID50 (F); the survival of mice was observed and recorded daily (* P<0.05, Log‐rank test) (G). H) JEV NS1‐Flag and WWP2‐Myc/shWWP2 (1 µg) plasmids were co‐transfected in 293T cells and treated with MG132 (5 µM) for 4 h after 48 h. NS1‐Flag was immunoprecipitated, and ubiquitination of JEV NS1‐Flag protein was detected by Western Blot. I) JEV NS1‐Flag and WWP2‐Myc/shWWP2 (1 µg) plasmids were co‐transfected in 293T cells, and the cells were collected after 48 h. NS1 protein levels were detected by Western Blot. J) JEV NS1‐Flag and WWP2‐Myc were co‐transfected in 293T cells, which were treated with MG132 (5 µM) for 4 h after 24 h. JEV NS1 protein levels were detected by Western Blot. K‐N) Cells were infected with LGTV after overexpression or knockdown of WWP2 in 293T (K) and U3A cells (M) (MOI = 1), and cellular RNA was extracted after 48 h. The viral RNA load in the cells was detected by using qRT‐PCR; etch‐a‐sketch assay was performed to detect the amount of infectious virus in the supernatants of U3A cells (N). Data are representative of 3 independent experiments and presented as mean ± SD. *P < 0.05, **P < 0.01, and ***P < 0.001 (Student's t‐test).

To assess the conservation of the K265 and K284 loci of NS1 among other arthropod‐borne flaviviruses, we conducted multiple sequence comparisons using the Clustal Omega software. The results indicated that the 284 sites of ZIKV, JEV, YFV, DENV‐1, DENV‐2, and WNV NS1 were lysine (K), serine (S), or threonine (T), and all of the aforementioned amino acids are susceptible to ubiquitination^[^ ^37^ ^]^ (**Figure** **8**A). This observation suggests a degree of conservation at amino acid position 284 among these arthropod‐borne flaviviruses.

To explore whether WWP2 exerts a regulatory effect on other arthropod‐borne flaviviruses, we investigated its impact on the infectivity of JEV viruses. Following the overexpression or knockdown of WWP2 in 293T cells (Figure 8B) and interferon system‐deficient U3A cells (Figure 8C,D), we infected the cells with JEV and measured viral infection using qPCR and plaque assays. The results demonstrated a significant reduction in viral replication in cells after the overexpression of WWP2 and an increase in viral infection after knocking down WWP2. These data suggest that WWP2 can inhibit JEV infection and operates independently of the interferon system.

To further validate the inhibitory effect of WWP2 on JEV infection, we intraperitoneally injected WT and *Wwp*2^−/−^ mice with 10^7^ PFU of JEV (SA14). The amount of JEV in the blood cells (Figure 8E) and the viral titer in mouse serum on day 5 post‐infection (Figure 8F) were detected. The results indicated a significant increase in JEV load in *Wwp*2^−/−^ mice compared to WT mice. Moreover, *Wwp* 2^−/−^ mice infected with JEV exhibited a substantial increase in mortality (Figure 8G), further confirming the role of WWP2 in inhibiting JEV infection.

We then investigated whether the inhibition of JEV by WWP2 was accomplished by influencing NS1 protein levels. The results revealed that the overexpression of WWP2 significantly enhanced the ubiquitination of JEV NS1 (Figure 8H), while the knockdown of WWP2 reduced the ubiquitination of JEV NS1 (Figure 8H). This indicates that WWP2 can catalyze the ubiquitination of JEV NS1 protein. Furthermore, the overexpression of WWP2 dose‐dependently down‐regulated JEV NS1 protein levels, whereas the knockdown of WWP2 significantly upregulated NS1 protein expression (Figure 8I). Notably, WWP2 lost its down‐regulatory effect on JEV NS1 protein in the MG132‐treated group (Figure 8J). This result suggests that WWP2 also mediates the ubiquitination and degradation of JEV NS1.

To further elucidate the role of WWP2 in other arthropod‐borne flavivirus infections, we investigated its regulatory impact on a tick‐borne flavivirus, LGTV. Cells were infected with LGTV after the overexpression or knockdown of WWP2 in 293T cells (Figure 8K,L) and U3A cells (Figure 8M,N). The results of qRT‐PCR and virus titration assays demonstrated that WWP2 could inhibit LGTV infection, indicating that WWP2 can hinder various arthropod‐borne flavivirus infections.

### 2.8 Ubiquitination of NS1 by WWP2 Homologs in Mosquitoes Promotes ZIKV Infection of Mosquitoes

**Figure 9:**
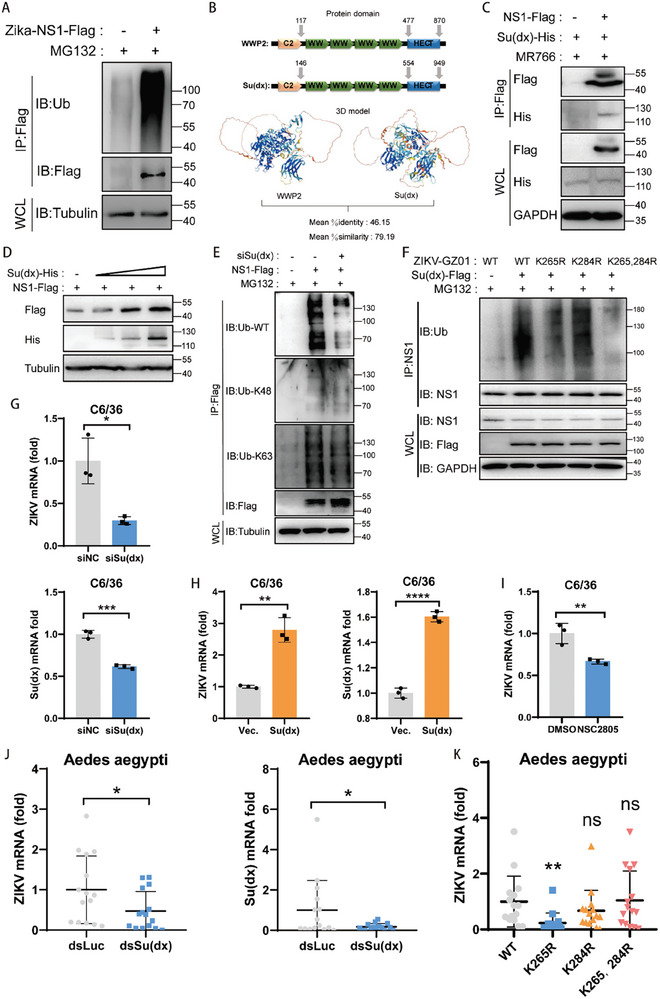
Ubiquitination of NS1 by WWP2 homologs in mosquitoes promotes ZIKV infection of mosquitoes. A) ZIKV NS1‐Flag was transfected in C6/36 cells and treated with MG132 (5 µM) for 4 h after 24 h. Cells were collected and immunoprecipitated with Flag antibody‐coupled magnetic beads, and the ubiquitination level of viral proteins was detected by Western Blot. B) The E3 ligase Su(dx), which is highly homologous to human WWP2, is present in Aedes albopictus. (WWP2 GenBank: U96114.2; Su(dx) GenBank: XM_01 969 6185.2) C) NS1‐Flag was co‐transfected with Su(dx)‐His expression plasmid in C6/36 cells and infected with ZIKV (MR766) (MOI = 0.5) 24 h later for 24 h. NS1‐Flag was immunoprecipitated, and Su(dx)‐His protein was detected by Western Blot. D) NS1‐Flag and different doses of Su(dx) were co‐transfected in C6/36 cells, and NS1‐Flag protein levels were detected by Western Blot 24 h later. E) Co‐transfected siSu(dx) (50 nM) with NS1‐Flag (1 µg) in C6/36 cells, treated with MG132 (5 µM) for 4 h after 24 h. Immunoprecipitation of NS1‐Flag was performed, and the ubiquitination level of NS1 protein was detected by Western Blot method. F) Su(dx)‐Flag (1 µg) was transfected into C6/36 cells. After 24 h, the cells were infected with WT, K265R, K284R and K265, 284R viruses (MOI = 0.5), respectively. 48 h later, the cells were treated with MG132 (5 µM, 4 h) and NS1 was immunoprecipitated. NS1 protein was detected by Western Blot and its ubiquitination level was determined. G) Transfection of siSu(dx) (50 nM) in C6/36 cells was followed by infection with ZIKV (MOI = 1) after 48 h. Viral mRNA levels in the cells, as well as Su(dx) knockdown efficiency, were detected after 24 h using qRT‐PCR. H) Su(dx)‐His was transfected in C6/36 cells, infected with ZIKV (MOI = 1) 24 h later, and the viral mRNA level as well as the efficiency of Su(dx) overexpression was detected in the cells 48 h later using qRT‐PCR. I) NSC2805 (10 µM, 4 h) treated C6/36 cells were infected with ZIKV and viral RNA levels were detected by qRT‐PCR at 24 h J) Aedes aegypti mosquitoes were divided into two groups, the experimental group was injected with Su(dx) dsRNA, and the control group was injected with Luc dsRNA. 100 PFU of MR766 strain virus was injected into each mosquito. The viral mRNA level and Su(dx) knockdown efficiency in mosquitoes were detected by qRT‐PCR on day 7 after infection. K) Recombinant viruses (WT, K265R, K284R, and K265/284R) of the same titer were injected into the thoracic cavity of Aedes aegypti mosquitoes (50 PFU of virus per mosquito), and viral loads in the mosquitoes were detected by qRT‐PCR on day 7 after infection. Data are representative of 3 independent experiments and presented as mean ± SD. ns, non‐significant, * *P* < 0.05, **P < 0.01, and ***P < 0.001, ****P < 0.0001 (Student's t‐test).

To validate whether the above results also occur in the mosquito vector, we investigated the ubiquitination status of the ZIKV NS1 protein in mosquito cells (C6/36). NS1 protein was overexpressed in C6/36 cells, and the ubiquitination level of NS1 was assessed using a Ub antibody following immunoprecipitation of NS1. The results revealed that NS1 also undergoes significant ubiquitination in C6/36 cells (**Figure** **9**A).

Our subsequent investigation aimed to determine whether a homologous counterpart of WWP2 also mediates modifications in the mosquito vector. We identified that the mosquito Su(dx) gene exhibits a high sequence homology with mammalian WWP2 (Figure 9B). To validate this finding, we conducted immunoprecipitation experiments, and the results demonstrated a direct interaction between NS1 and Su(dx) (Figure 9C). Surprisingly, Su(dx) had no significant effect on the degradation of the viral protein NS1 (Figure 9D). Furthermore, we analyzed the specific types of ubiquitination of NS1 regulated by Su(dx) using endogenous ubiquitination antibodies. We found that the K63 type of ubiquitination of NS1 proteins was significantly decreased by the knockdown of Su(dx), whereas there was no effect on the K48 type of ubiquitination (Figure 9E). To identify the specific site on NS1 ubiquitinated by Su(dx) in mosquitoes, we overexpressed Su(dx)‐Flag in C6/36 cells infected with WT, K265R, K284R, and K265,284R viruses. The ubiquitination levels were assessed by immunoprecipitation using NS1. The experimental results indicated a significant reduction in NS1 ubiquitination upon mutation at the K265 locus, suggesting that Su(dx) targets the Lys 265 site for ubiquitination (Figure 9F). These findings suggest that Su(dx) mediates K63‐type ubiquitination of the NS1 Lys 265 site in mosquito cells.

Given that mammalian WWP2 exerts an antiviral effect in the host, we investigated the impact of Su(dx) on ZIKV infectivity in C6/36 cells. Interestingly, the viral load in the cells significantly increased after the overexpression of Su(dx) compared with the control group, while the viral load decreased after the knockdown of intracellular Su(dx) expression (Figure 9G,H). NSC2805, a natural chemical inhibitor of WWP2, inhibited viral replication in C6/36 cells (Figure 9I). We further silenced the Su(dx) gene using dsRNA‐mediated RNAi and infected *Aedes aegypti* with ZIKV through a microinjection mode. The results demonstrated that the knockdown of Su(dx) led to a significant reduction in the viral load of ZIKV (Figure 9J). These findings suggest that Su(dx) promotes ZIKV infection in the mosquito vector, contrary to its role in the host. Finally, we infected mosquitoes with recombinant viruses packaged by reverse genetics and observed that the viral load of infected K265R viruses was significantly lower than that of WT viruses (Figure 9K). This phenomenon suggests that the mutation in the K265R locus of NS1 reduces the infection efficiency of the virus in mosquito vectors, and this result may partially explain why K265 viruses become predominantly endemic in nature.

## 3 Discussion

Based on the above experimental results, we observed an up‐regulation of WWP2 expression during ZIKV infection or IFN stimulation, highlighting WWP2 as an interferon‐inducible gene (ISG). Interferons play a pivotal role in the innate immune response by inducing the expression of numerous ISGs. However, distinct ISGs may be involved in restricting infections by different types of viruses.^[^ ^38^ ^]^Through in vivo and in vitro experiments, we established that WWP2 inhibits ZIKV infection independently of the interferon system. This suggests that the WWP2‐mediated ubiquitination and degradation of viral proteins may represent an inducible antiviral mechanism in the mammalian host.

WWP2 functions as an E3 ubiquitin ligase, catalyzing the ubiquitination of target proteins and thereby influencing protein stability.^[^ ^32^, ^33^, ^39^ ^]^Typically, polyubiquitinated proteins undergo ubiquitin linkage via K48 or K63. K48‐linked ubiquitination is commonly associated with protein degradation, while K63 modification often impacts protein function and localization. Intriguingly, our findings revealed that both K63 and K48 types of ubiquitination at the K265 and K284 sites on the NS1 protein contribute to its degradation in mammalian cells. Previous studies have reported that the E3 ubiquitin ligase WWP1 catalyzes K63‐linked ubiquitination of mHtt, inhibiting mHtt degradation through the ubiquitin‐proteasome pathway.^[^ ^40^ ^]^However, emerging evidence also suggests that K63‐type ubiquitination can play a role in protein degradation.^[^ ^41^ ^]^

Through reverse genetics techniques, we discovered that NS1 mutant viruses with K265R, K284R, and K265/284R double mutations exhibit higher infectivity in mammalian host compared to WT viruses. Importantly, the K265R, K284R mutation diminishes the regulatory influence of WWP2 on ZIKV. Our observations align with a study by Peiyong Shi et al., where they reported that the ZIKV NS1 K265E mutation significantly enhances viral replication in Vero E6 cells.^[^ ^42^ ^]^They propose that the NS1 K265E mutation augments the NS1/NS2A interaction, facilitating virus assembly. This implies that the K63‐type ubiquitination observed in NS1 K265 not only relates to protein degradation but may also play a role in regulating virus assembly. Our previous data indicated higher secretion of the mutant NS1 compared to the WT virus. We hypothesize that the K265R and K284R mutations may impact the stability of the hexameric form of NS1 or its secretion, in addition to enhancing the overall stability of the K265R and K284R mutant NS1 in mammals.

Our investigation, aided by a search through viral sequence libraries, revealed the existence of a natural ZIKV NS1 mutant virus strain at amino acid positions 265/284, adding significance to our study. Despite the presence of these ZIKV NS1 265/284 amino acid mutant strains in nature, they have not led to large‐scale epidemiologic spread. We propose that this phenomenon may be attributed to selective pressure on these two ubiquitination sites exerted by both the host and the mosquito vector during ZIKV infection. As an arbovirus, the processes of virus acquisition, replication, and transmission in vectors are crucial. Evidence shows that ubiquitination also regulates arbovirus infection in mosquito vectors.^[^ ^23^, ^24^, ^25^, ^26^, ^27^ ^]^Our preliminary exploration of NS1 ubiquitination in mosquitoes showed that NS1 undergoes ubiquitination mediated by Su(dx), an E3 ligase in mosquitoes homologous to WWP2 in mammals. Unexpectedly, Su(dx)‐mediated ubiquitination is predominantly of the K63‐type and does not lead to NS1 degradation but rather enhances NS1 stability. The differing roles of NS1 Lys 265 in hosts and vectors were also observed in a study by Peiyong Shi et al., where they reported that the NS1 K265E mutation enhanced viral replication in mammalian cells while attenuating viral infection in mosquitoes.^[^ ^42^ ^]^This observation may provide insight into why K265 viruses are predominantly endemic strains in nature.

Protein homeostasis is intricately maintained through a dynamic interplay of ubiquitination and deubiquitination processes, where deubiquitinating enzymes can counteract the effects of E3 ligases by removing ubiquitin moieties from substrates. In our exploration of deubiquitinating enzymes acting on NS1 proteins, mass spectrometry revealed that USP10 interacts with NS1. Subsequent experiments demonstrated that USP10 catalyzes the deubiquitination of NS1, thereby enhancing NS1 protein stability. Correspondingly, USP10 was found to promote ZIKV infection (Figure S4, Supporting Information). This suggests that the virus may employ this strategy to evade host ubiquitination and prevent the degradation of NS1. These findings contribute to a more comprehensive understanding of the intricate roles of NS1 protein ubiquitinations during ZIKV infection in mammalian hosts.

Recently, Zu et al. found that TRIM22 acts as an E3 ubiquitin ligase and degrades NS1 and NS3 proteins by ubiquitination, thereby inhibiting the infection of other flaviviruses such as ZIKV, DENV and YFV.^[^ ^29a^ ^]^Although this conclusion was validated in in vitro experiments, the effect of TRIM22 on flavivirus infection in vivo has not been further explored. Ji et al. found that TRIM22 affects viral replication by regulating intrinsic immunity,^[^ ^43^ ^]^so the inhibition of flavivirus infection by TRIM22 may also be achieved by modulating innate immune signaling pathways. Moreover, Zu et al. have no clear evidence for the sites and types of ubiquitination of NS1 and NS3 proteins catalyzed by TRIM22, so the mechanism by which TRIM22 regulates flavivirus infection remains to be explored.

Our investigation revealed that WWP2 exhibits inhibitory effects on flaviviruses such as JEV and LGTV, substantiating its role as a pan‐restriction factor for arthropod‐borne flaviviruses. Given the similarities among these viruses, it is common for them to be regulated by similar mechanisms. For instance, TRIM5α impedes flavivirus replication by targeting the viral protease NS2B3, thereby inhibiting viral replication;^[^ ^8^ ^]^ Similarly, the interferon‐stimulated gene Slfn11 hampers flavivirus replication, encompassing WNV, DENV, and ZIKV;^[^ ^44^ ^]^ Furthermore, Akt kinase disrupts flavivirus replication by interacting with the viral protein NS5.^[^ ^45^ ^]^Given that LGTV shares more than 80% amino acid homology with TBEV and is a natural attenuated member of the TBEV serogroup,^[^ ^46^ ^]^we postulate that WWP2 may also inhibit TBEV infection ‐a hypothesis that warrants further verification.

The classical ubiquitination pathway typically involves the attachment of a ubiquitin molecule to a lysine residue of a target protein. However, ubiquitin can also be linked to serine, threonine, and tyrosine via hydroxyphosphate bonds, constituting a non‐classical ubiquitination pathway. An illustrative example of this pathway is observed in Bid proteins, which undergo degradation in the apoptotic pathway.^[^ ^47^ ^]^Non‐classical ubiquitination has been associated with the endoplasmic reticulum‐associated degradation (ERAD) pathway. Emerging evidence suggests that non‐classical ubiquitination in ERAD is upregulated during various viral infections. Our preliminary data indicate that WWP2 does not significantly affect DENV infectivity. Considering that amino acid 284 of DENV NS1 is a threonine, we speculate that WWP2 may catalyze this site for non‐classical ubiquitination. The relatively weak stability of the ester bond formed between ubiquitin and threonine could be one factor contributing to why WWP2 does not regulate DENV.

In conclusion, WWP2 emerges as a broad‐spectrum restriction factor against arthropod‐borne flaviviruses, inhibiting ZIKV, JEV, and LGTV infections in mammals. Numerous small‐molecule natural products have been developed as agonist analogs to enhance the expression of E3 ubiquitin ligases, contributing significantly to the development of novel drugs for various diseases.^[^ ^48^ ^]^This approach holds promise in offering innovative perspectives for the development of antiviral drugs.

## 4 Experimental Section

### Viruses, Cells and Mice

ZIKV was propagated in C6/36 cells (*Aedes albopictus* cells) and titrated using Vero E6 (monkey kidney cell line); JEV was propagated and titrated using Vero E6 cells. LGTV, kindly provided by Dr. Zhenhua Zheng at the Wuhan Institute of Virology, was proliferated on BHK21 cells (hamster kidney fibroblasts), and titrated using Vero E6 cells.

293T (human embryonic kidney cell line), Vero E6, SH‐sy5y (human neuroblastoma cells), BHK21, U3A (STAT1‐deficient human fibrosarcoma cells) celllines were cultured using DMEM medium with 10% fetal bovine serum in a thermostatted cell culture incubator at 37 °C 5% CO_2_; C6/36 were cultured using MEM medium with 10% fetal bovine serum at 28 °C 5% CO_2_ in a thermostatic cell culture incubator.

Interferon receptor‐deficient (*Ifnar1^−/−^*) C57BL/6 mice were gifted by Prof. Chunsheng Dong at Soochow University. C57BL/6 mice with WWP2 deletion (*Wwp 2^−/−^*) genotype were purchased from Gempharmatech Co. (Nanjing, China) and were housed in an SPF‐grade animal facility at Soochow University. Laboratory animal welfare and laboratory licenses are in accordance with the National Laboratory Animal Health Guidelines. Animal experiments were approved by the ethics committee of Soochow University and Suzhou Institute of Systems Medicine.

### Antibodies

The following antibodies were used in this study: GAPDH Mouse Antibody (Proteintech, Cat # 60004‐I‐Ig), DYKDDDDK Tag Mouse Antibody (ABclonal, Cat # AE005), anti‐rabbit IgG HRP‐linked antibody (CST, Cat # 7074), HRP goat anti‐mouse IgG (BioLegend, Cat # 405 306), HA‐tag Rabbit Polyclonal Antibody (CST, Cat # 3724), Ubiquitin Antibody, (Santa Cruz, Cat # sc‐8017), K63‐linkage Specific Polyubiquitin (D7A11) Rabbit mAb (CST, Cat # 12 930), K48‐linkage Specific Polyubiquitin (D9D5) Rabbit mAb (CST, Cat # 12 805), CACYBP Polyclonal Antibody (Abclonal, Cat # A8757), TRIM4 Polyclonal Antibody (Abclonal, Cat # A15922), WWP2 Polyclonal Antibody (Abclonal, Cat # 12197‐1‐AP), Myc‐tag Rabbit Polyclonal Antibody (Proteintech, Cat # I6286‐I‐AP), Goat Anti‐Mouse IgG Antibody (H+L), DyLight 488 (SeraCare, Cat # 5230‐0391), Goat Anti‐Rabbit IgG H&L (Alexa Fluor 647) (abcam, Cat # ab150083), GST Tag Antibody (ABGENT), ZIKV virus NS1 protein antibody (GeneTex, Cat # GTX133307, GTX634158).

### Reagents

Agilent Lightning Site‐Directed Mutagenesis Kit (Agilent Technologies, Cat # 200 518), Ribomax SP6 large‐scale RNA production kit (Promega, Cat # P1280), T7 High Efficiency Transcription Kit (TransGen Biotech, Cat # JT101‐01), Transfection reagent LongTrans (UCallM), MG132 (Selleck, Cat # S2619), Chloroquine (MCE, Cat # HY‐17589A), 2×Taq Master Mix (Novoprotein, Cat # E005‐01), 2×Phanta Max Master Mix, (Vazyme, Cat # P525‐01), Polybrene (Yeasen, Cat # 40804ES76), Protein A/G immunomagnetic beads (Bimake, Cat # B23201), Anti‐Flag immunomagnetic beads (Bimake, Cat # B26101), Glutathione Beads 4FF (Smart‐Lifesciences, Cat # SA010025), E3 Ligase Auto‐Ubiquitylation Assay Kit (Abcam, Cat # ab139469), NSC2805 (MedChemExpress, Cat # HY‐134417).

### Plasmids

The open reading frames of specific viral and host genes were subcloned into plasmids with epitope tags: Flag‐ZIKV‐C, Flag‐ZIKV‐PrM/E, Flag‐ZIKV‐NS1, Flag‐ZIKV‐NS2A, Flag‐ZIKV‐NS2B, Flag‐ZIKV‐NS3, Flag‐ZIKV‐NS4A, Flag‐ZIKV‐NS4B, Flag‐ZIKV‐NS5,. Flag‐JEV‐NS1, Myc‐TRIM4, Myc‐CACYBP, Myc‐HUWE1‐HECT, Myc‐STUB1, Myc‐Trim13, Myc‐UBR5‐HECT, Myc‐WWP1, Myc‐ZNF598, Myc‐WWP2, Myc‐Su(dx), GST‐WWP2, GST‐Su(dx), Flag‐ZIKV‐NS1, His‐Su(dx), and His‐WWP2. HA‐Ub, HA‐K11, HA‐K48, HA‐K63, were obtained from Addgene. HA‐K6, HA‐K27, HA‐K29, and HA‐K33 were kindly donated by Prof. Hui Zheng of Soochow University. shCACYBP, shTRIM4, shWWP2, shmWWP2: insertion of targeting sequences into the Lenti‐U6‐shRNA‐GFP‐puro vector. pACNR‐GZ01‐Intron‐IC is kindly provided by Prof. Chengfeng Qin, Academy of Beijing Medical Sciences, China.

### Western Blotting

Cell lysis buffer and protease inhibitor were added to the cells and lysed on ice for 20 min. Centrifugation was performed at 4 °C, 12 000 rpm for 10 min. The bottom precipitate was removed, and the protein supernatant was pipetted into a new EP tube. SDS‐PAGE protein loading buffer was added and placed in a metal bath at 100 °C for 10 min. The protein supernatant was then loaded to a 10% SDS‐PAGE gel, followed by electrophoresis, wet transfer, closure, and incubation with antibodies. Finally, it was developed using a fully automated digital gel/chemiluminescence image analysis system instrument.

### Immunoprecipitation

Add an appropriate amount of antibody and magnetic beads to the prepared protein supernatant and incubate at 4 °C for 8–12 h. The mixture of protein samples and magnetic beads was placed on a magnetic rack, the supernatant was discarded, and the beads were washed three times with TBST for 1 min each time. Proteins were eluted from the magnetic beads using an elution solution or added to protein loading buffer and placed in a metal bath at 100 °C for 10 min. The obtained protein samples were used for subsequent experiments, including western blotting, GST pull‐down, or mass spectrometry.

### Viral Titrations

A uniform layer of Vero E6 cells was spread in a 96‐well cell culture plate, and the viral solution was gradient diluted (10^−1^ to 10^−10^) with culture medium. One hundred microliters of the diluted virus were added to the Vero E6 cells individually. On day 3–5, the cell cytopathic effects (CPE) were recorded. The viral titer was calculated using the Reed‐Muench two‐component or Karber methods.

Plaque Assay: Serial dilutions of viral supernatants were added to BHK21 cells in 24‐well plates. After incubation at 37 °C for 2 h, the viral solution was discarded, and the infected cells were overlayed with 1 ml of medium containing 2% FBS and 0.5% low melting point agarose (Promega, USA) and incubated for another 5 days. The cells were then fixed with 4% formaldehyde and stained with 1% crystal violet dissolved in 20% ethanol. Photographs of the plaques were taken with a digital camera.

### qRT‐PCR

Cellular RNA was extracted using Total RNA Kit I (Omega, Norcross, USA). The RNA was reverse‐transcribed to generate cDNA using HiScript Q RT SuperMix for qPCR (Vazyme, Cat # R122‐01). The mRNA expression levels of the tested genes were analyzed by real‐time quantitative PCR using PerfectStartTM Green qPCR SuperMix (Transgen, Cat # AQ601).

### Cellular Infection with Lentivirus

293T cells were cultured in Cell culture dishes and transfected with helper plasmids psPAX2, pMD2G, and shmWWP2 when the density was ≈70%. After 48 h, the supernatant was centrifuged at 4 °C, 4000 rpm for 10 min to harvest the lentivirus. The lentiviral supernatant was added to the cells along with polybrene (final concentration of 0.1 µg ml^−1^) and incubated at 37 °C for 72 h. Subsequently, ZIKV infection was introduced for the next step of the experiment, following observation by fluorescence microscopy.

### Viral Infection of Mice

Lentiviral shmWWP2 was administered to *Ifnar1*‐deficient C57BL/6 mice via the tail vein route, followed by an intraperitoneal injection of 10^7^ PFU ZIKV virus seven days later. Blood samples were collected on the 3rd and 5th days after infection, and mouse mortality was observed daily. Tissues were collected after euthanizing the mice, and qRT‐PCR was used to detect the viral load in mouse whole blood cells.

WT mice and *Wwp2* ^−/−^ mice were intraperitoneally injected with 10^7^ PFU JEV virus. Blood samples were collected on the 3rd and 5th days after infection, and mortality was observed. qRT‐PCR was performed to detect the viral load in mouse whole blood cells.

### Prokaryotic Protein Expression and Purification

The ORF encoding WWP2 was inserted into the pEGX‐6P‐2 vector. The WWP2‐GST protein underwent expression and purification in *E. coli* BL21 (DE3). GST‐tagged WWP2 was induced into a soluble form by treating the *E. coli* culture with 0.1 mM isopropyl β‐d‐thiogalactopyranoside for 4 h at 25 °C. The resultant recombinant fusion proteins were subsequently subjected to incubation with GST affinity agarose (GE Healthcare, Sweden).

### In Vitro Ubiquitination Assay

The in vitro ubiquitylation assay was conducted using the E3 Ligase Auto‐Ubiquitylation Assay Kit (Abcam). Immunoprecipitated NS1, prokaryotically purified recombinant WWP2‐GST, E1 (Hdm2), and E2 (UbcH5a) were incubated in the presence of ATP as per the provided instructions. Subsequently, western analysis was performed to assess the in vitro ubiquitination level of NS1.

### Immunofluorescence (IF) Analysis

293T cells were infected ZIKV; Or cells were co‐transfected with WWP2‐Myc and NS1‐Flag plasmids, followed by ZIKV infection. After fixing the cells in 4% formaldehyde for 10 min, they were sequentially permeabilized with 0.4% Triton X‐100, blocked with 5% FBS, and incubated overnight at 4 °C with primary antibodies (rabbit anti‐flag and mouse anti‐myc). Subsequently, TRITC‐Goat anti‐mouse IgG (H+L) (Jackson) and Goat anti‐Rabbit IgG‐FITC (Southern Biotech) were applied for 1 h at room temperature. Cell nuclei were stained with DAPI, and images were captured using confocal microscopy.

### Enzyme‐Linked Immunosorbent Assay (ELISA)

Inactivated viral supernatants were immobilized on enzyme‐linked immunosorbent plates (Costar) using encapsulation buffer (Biolgend) for 16 h at 4 °C. The plates were washed three times with PBST (0.1% Tween 20 in PBS; pH 7.4) and blocked with 1% BSA (BBI Life Sciences) in PBS for 1 h at room temperature. After washing, the plates were incubated with a primary antibody for 1 h and then with an enzyme‐labeled secondary antibody for an additional 1 h. Subsequently, TMB solution (Thermo Scientific) was added to the plates. The reaction was halted by adding 1 M H_2_SO_4_, and the optical density at 450 nm (OD450) was measured using a Bio‐Tek Instruments universal enzyme marker.

### Sample Preparation for Mass Spectrometry

Preparation of whole‐cell protein samples for protein profiling were performed according to standard protocols^[^ ^49^ ^]^: SH‐sy5y cells were infected with ZIKV at an MOI of 1. Cell precipitates were collected at 48 h post infection. The cell lysate, containing 8 M urea and protease inhibitors, was sonicated in an ice bath. Dithiothreitol (DTT, Sigma) was added to 200 µg of protein supernatant at a final concentration of 5 mM and incubated at 56 °C for 30 min. Iodoacetamide (IAA, Sigma) was then added to a final concentration of 11 mM and incubated in a light‐proof environment for 15 min. Enzymatic digestion was carried out by adding trypsin (Thermo Fisher) and incubating at 37 °C for 16 h. The enzyme solution was dried in a vacuum desiccator, re‐solubilized with 0.1% formic acid solution, and desalted using a MonoSpin C18 protein desalting column (GL Sciences). Peptides were dissolved in 10 µl of 0.1% formic acid.

The NS1‐interacting proteins were identified by mass spectrometry: 293T cells were transfected with FLAG‐NS1 or FLAG empty vector. At 24 h post‐transfection, cells were immunoprecipitated with anti‐FLAG‐agarose. After Coomassie Brilliant Blue staining, excised gel segments were subjected to in‐gel trypsin digestion and dried. Peptides were dissolved in 10 µl 0.1% formic acid. Peptides were characterized by the liquid chromatography‐mass spectrometry (LC‐MS/MS). The raw data using the Proteome Discoverer 2.2 proteomics analysis software platform was analyzed.

### Molecular Docking

The 3D structures of the WWP2‐WW structural domain (PDB ID: 6rss) and ZIKV NS1 protein (PDB ID: 5 × 8y) were obtained from the Protein Data Bank (PDB). The binding sites of the two proteins were predicted using GRAMM with default parameters, and the docking results were visualized using PyMOL software.

### Mosquitoes Infection with ZIKV

*Aedes aegypti* mosquitoes, aged 5–7 days post‐pinning, were starved for 16–24 h. After cryoanesthesia, microinjections were performed on mosquitoes under a microscope, and each mosquito was injected with 100 ng of dsRNA and 100 PFU of ZIKV. Mosquitoes were placed in 750 ml paper cups and provided with 10% sucrose water for rapid recovery. The mosquitoes were kept at 28 °C and 70% relative humidity. On day 7 post‐infection, mosquitoes were collected and stored at −80 °C overnight before RNA extraction.

### GST‐Pull Down

The expression of WWP2‐GST was induced by IPTG (1 mM) in *Escherichia coli* BL21(DE3). Then, the recombinant fusion proteins were incubated with GST affinity agarose separately. Thereafter, ectopic expressed NS1‐Flag purified from 293T cell lysate was incubated with the agarose, and the immunoprecipitated proteins were analyzed by western blot analysis.

### Histological Analysis

Brain tissues from control or ZIKV‐infected mice were fixed in 4% formaldehyde solution and embedded in paraffin. Paraffin sections were stained with a hematoxylin–eosin solution, and histological changes were observed using a light microscope (Nikon, Tokyo, Japan).

### Statistical Analysis

The experimental data were processed, plotted, and analyzed using GraphPad Prism 8 software. Statistical analyses were performed using ANOVA, *t*‐test, and Log‐rank test, respectively. Symbols indicate significance levels: *P < 0.05, **P < 0.01, ***P < 0.001, ****P < 0.0001; ns: no significance.

### Ethical Statement

Laboratory animal welfare and laboratory licenses are in accordance with the National Laboratory Animal Health Guidelines. Animal experiments were approved by the ethics committee of Soochow University and Suzhou Institute of Systems Medicine.

## Conflict of Interest

The authors declare no conflict of interest.

## Author Contributions

C.H., T.J., and W.P. contributed equally to this work. F.M. and J.D. performed conceptualization; C.H., T.J., W.P., T.F., Q.W., and X.Z. performed methodology; C.H., T.J., T.F., Q.W., and J.D. performed investigation; C.H. and J.D. performed formal analysis; C.H. and J.D. wrote the original draft; C.H., W.P. and J.D. wrote, reviewed and edited; C.H. and J.D. performed visualization; J.D. and F.M. performed funding acquisition; J.D. provide resources; J.D performed supervision.

## Supporting information

Supporting Information

## Data Availability

The data that support the findings of this study are available from the corresponding author upon reasonable request.
