# Supplementary material for: Ubiquitination of NS1 Confers Differential Adaptation of Zika Virus in Mammalian Hosts and Mosquito Vectors
Source: Adv Sci (Weinh). 2024 Aug 19;11(39):2408024. doi: 10.1002/advs.202408024 (PMC11497017; doi:10.1002/advs.202408024)

Supplementary Materials for

**Ubiquitination of NS1 confers differential adaptation of Zika virus in mammalian hosts and mosquito vectors**

Chenxiao Huang *et al.*

*Corresponding author. Email: daijianfeng@suda.edu.cn

**This PDF file includes:**

Supplementary Figure S1-S4 and the legends

Tables S1

Graphic Abstract

Supplmentary Figures and Legends


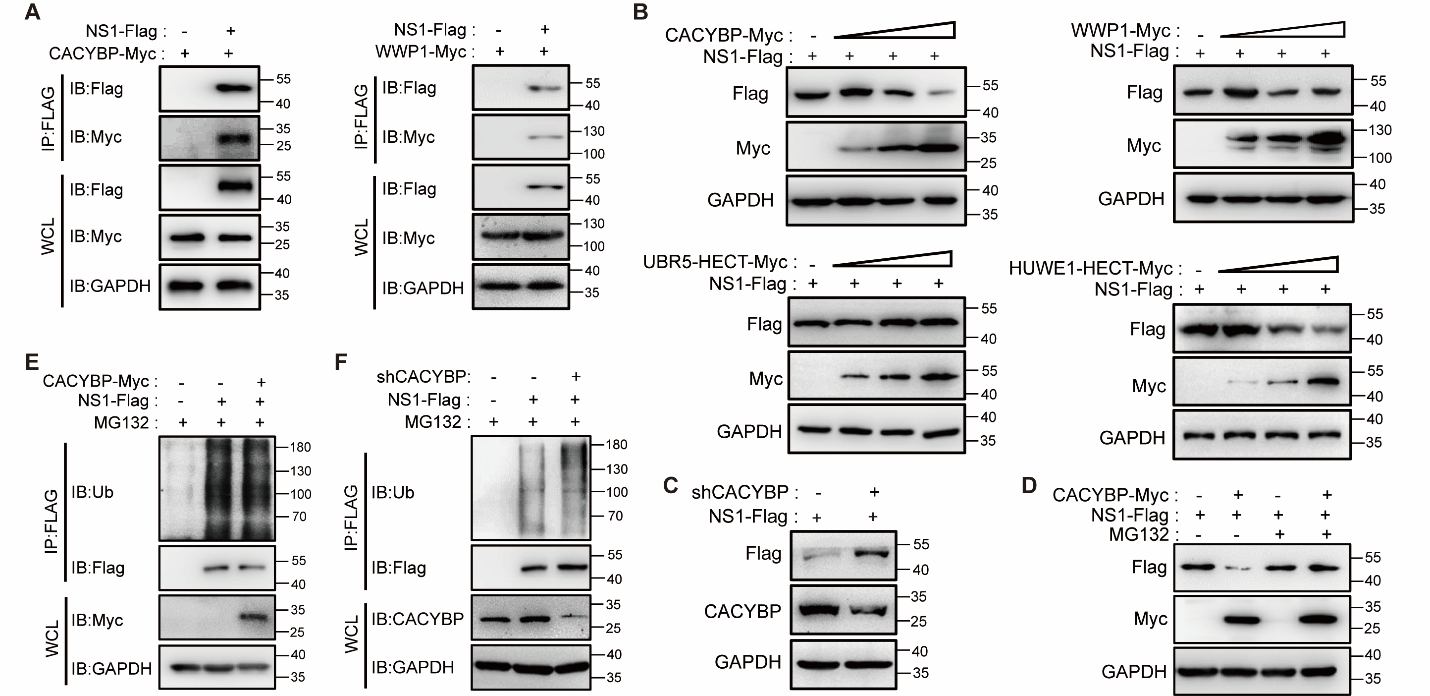


Figure S1: **Screening for NS1-related E3 ubiquitin ligase related proteins**

**A:** NS1-Flag was co-transfected with CACYBP-Myc and WWP1-Myc expression plasmids in 293T cells, NS1-Flag was immunoprecipitated 24 hours later, and CACYBP-Myc and WWP1-Myc proteins were detected by Western blotting.

**B:** NS1-Flag and different doses of CACYBP, WWP1, UBR5-HECT, or HUWE1-HECT were co-transfected in 293T cells, and NS1-Flag protein levels were detected by Western Blot 24 h later.

**C-D:** NS1-Flag and shCACYBP/CACYBP-Myc (1 μg) were co-transfected in 293T cells, , and NS1-Flag protein levels were detected by Western Blot 24 h later.

**E-F:** NS1-Flag and CACYBP-Myc/shCACYBP (1 μg) were co-transfected in 293T cells, which were treated with MG132 (5 μM) for 4 h after 24 h. NS1-Flag was immunoprecipitated, and the level of ubiquitination of NS1-Flag proteins was detected by Western Blot.

Data are representative of 3 independent experiments.


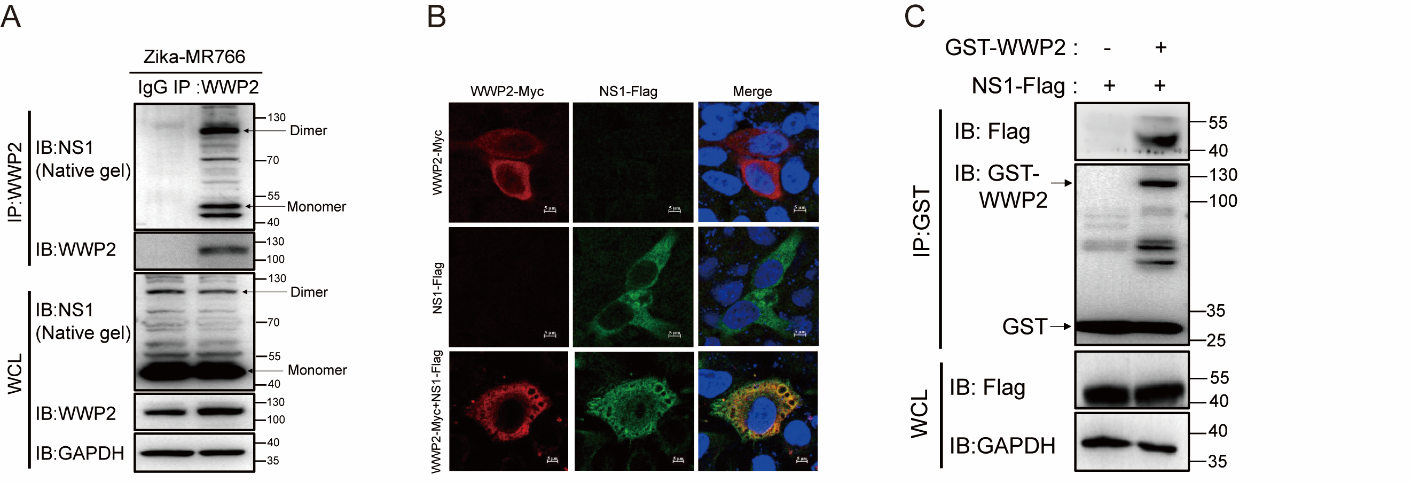


Figure S2: **WWP2 interacts with NS1**

A: 293T cells were infected with ZIKV (MOI=0.5) for 48 hours, and endogenous WWP2 was immunoprecipitated. ZIKV NS1 protein was then detected after a non-denaturing protein electrophoresis.

B: NS1-Flag was co-transfected with WWP2-Myc in HeLa cells for 24 hours, followed by ZIKV infection (MOI=0.5) for 24 hours. The intracellular localization of the two proteins was observed using laser confocal imaging.

**C:** GST-pulldown was performed to test the association between WWP2 and NS1. The expression of WWP2-GST was induced by IPTG (1 mM) in *Escherichia coli* BL21(DE3). Then, the recombinant fusion proteins were incubated with GST affinity agarose separately. Thereafter, NS1-Flag was incubated with the WWP2-GST and the immunoprecipitated proteins were analyzed by Western Blot.


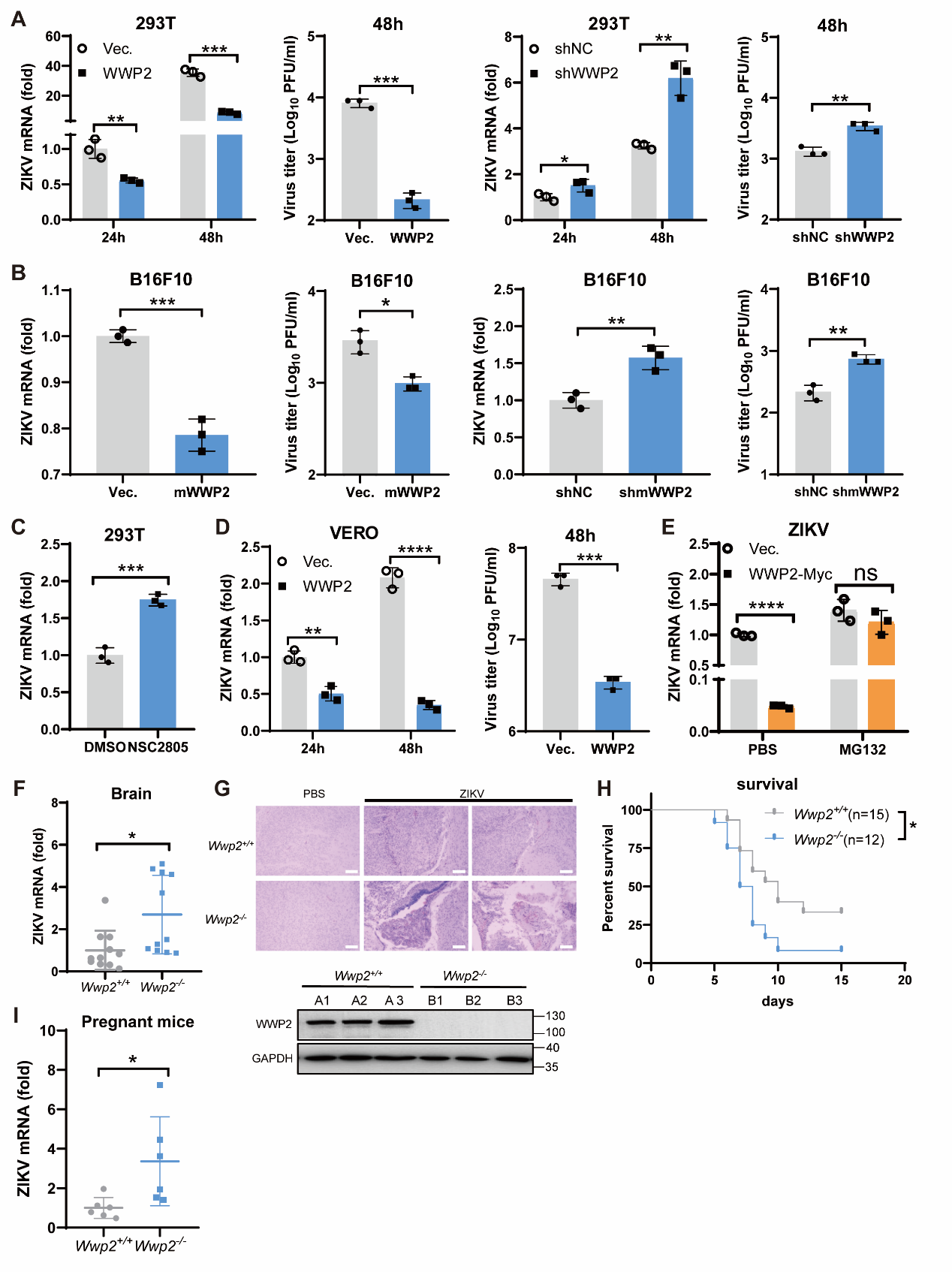


Figure S3: **WWP2 inhibits ZIKV infection in multiple cells**

**A-B:** WWP2 was overexpressed or knocked down in 293T/B16F10 cells and infected with ZIKV (MOI=0.5) for 48 hours. Cellular RNA was extracted at 24 h and 48 h, respectively, and qRT-PCR analyzed the amount of viral RNA replication. The titer of the viral supernatant after 48 h of infection was determined by TCID50. **C:** NSC2805 (10 μM, 4 h) treated 293T cells were infected with ZIKV and viral RNA levels were detected by qRT-PCR at 24 h.

**D:** WWP2 was overexpressed in Vero E6 cells and infected with ZIKV (MOI=0.5) for 48 hours. Cellular RNA was extracted at 24 h and 48 h, respectively, and qRT-PCR analyzed the amount of viral RNA replication. The titer of the viral supernatant after 48 h of infection was determined by TCID50.

**E**: WWP2 was overexpressed in 293T cells and infected with ZIKV (MOI=0.5) for 48 hours. After MG132 (5 μM, 4 h) treatment, the viral RNA levels were analyzed by qRT-PCR.

**F-H:** Using WT and *Wwp2 ^-/-^* mice, 20 PFU ZIKV was injected intracranial, and brain tissues were collected on days 3, respectively. RNA was extracted, and the amount of ZIKV in the brain was detected using qRT-PCR (F); Micrographs of hematoxylin-and-eosin-stained brain sections from mice treated with PBS or ZIKV. Scale bar, 100 μm (G); The survival of mice was observed and recorded daily (* *P*<0.05, Log-rank test) (H).

**I**: WT and *Wwp2^-/-^* pregnant mice were injected with 10^7 PFU ZIKV via the tail-vein route, Hemocytes were collected on days 3. Blood cell RNA was extracted, and qRT-PCR was used to detect the RNA content of ZIKV.

Data are representative of 3 independent experiments and presented as mean ± SD. ns, non-significant * *P*<0.05, ** *P* <0.01, and *** *P* < 0.001, **** *P*<0.0001 (Student’s t-test).


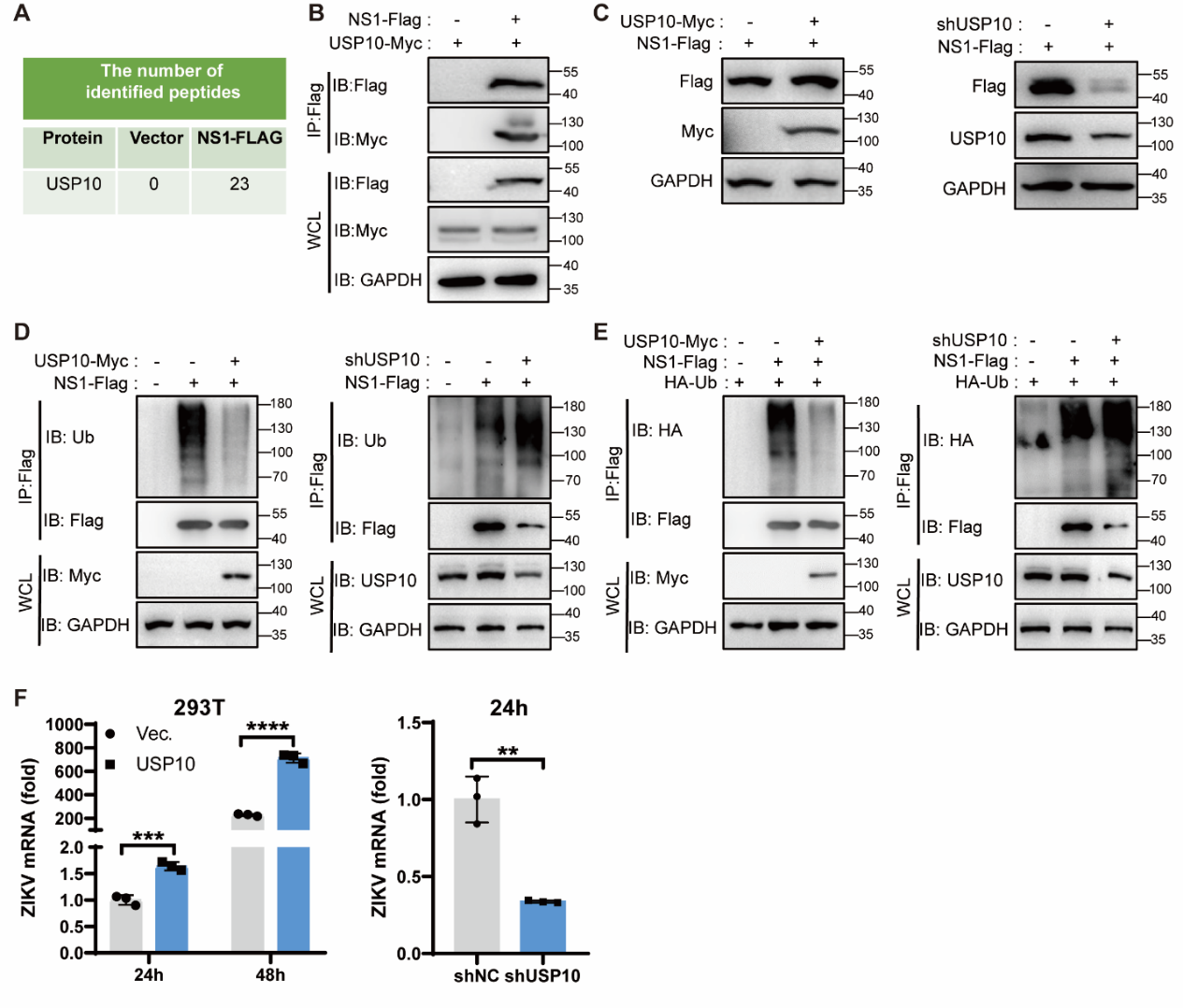


Figure S4: **USP10 is a deubiquitinating enzyme of ZKIV NS1**

**A-E:** Analysis of the interaction of NS1 with the deubiquitinating enzyme USP10 using mass spectrometry with immunoprecipitation (A and B); co-transfection of NS1-Flag and expression plasmids overexpressing or knocking down USP10 in 293T cells, and detection of the NS1-Flag protein level by Western Blot method (C); or detection of the NS1-Flag protein ubiquitination levels (D and E).

**F: Overexpression or knockdown of USP10 in 293T cells were infected with ZIKV** (MOI=0.5) after 48 h. Cellular RNA was extracted at 24 h and 48h, respectively, and qRT-PCR analyzed the amount of viral RNA replication. Data are representative of 3 independent experiments and presented as mean ± SD. ** *P* <0.01, and *** *P* < 0.001, **** *P*<0.0001 (Student’s t-test).

**Table S1:** **Sequences of oligo primers used in this stduy.**

| Primers used for qRT-PCR | |
| --- | --- |
| ZIKV E-Forward | CTGCCCAACACAAGGTGAA |
| ZIKV E-Reverse | ATGTCACCAGGCTCCCTTTG |
| JEV E-Forward | CACTGGACTGTGAGCCAAGGA |
| JEV E-Reverse | ACCCAAGAGCAACAACGGACT |
| LGTV E-Forward | TGGCAGGTGCATCGTGACT |
| LGTV E-Reverse | GCCTCAGCTCCATCATGCTT |
| Human WWP2-Forward | CCCCGAATCCCAACACGAC |
| Human WWP2 -Reverse | GTTCCCATCCAGCAGGCAGA |
| Mouse WWP2-Forward | AGGGCGTGCGGTACTTTGTG |
| Mouse WWP2-Reverse | GCAGAGGAAACGGAACTGGTGATA |
| Human-USP10-Forward | GAGAATGTAACCCTAATCCATAAACC |
| Human-USP10-Reverse | GCGGGCAAGCAACCAAT |
| Aedes aegypti actin-Forward | GAATGTGCAAGGCCGGTTTC |
| Aedes aegypti actin-Reverse | \| ATTGGGTACTTCAGGGTGAGGATA \| \| --- \| |
| Aedes albopictus actin -Forward | AGAAGGAAATCACCGCCCTG |
| Aedes albopictus actin -Reverse | GCTGGAAGGTGGATAGCGAG |
| Mouse GAPDH-Forward | GGCCTTCCGTGTTCCTACC |
| Mouse GAPDH-Reverse | AGCCCAAGATGCCCTTCAGT |
| Human actin-Forward | TGACGTGGACATCCGCAAAG 3' |
| Human actin-Reverse | CTGGAAGGTGGACAGCGAGG 3' |
| Aedes aegypti Su(dx) -Forward | CGAACGCAGGGTCAAGAGG |
| Aedes aegypti Su(dx) -Reverse | TGCACCAGGCCGTGGAT |
| Aedes albopictus Su(dx) -Forward | ATCCGTTACACCGCAACTGGG |
| Aedes albopictus Su(dx) -Reverse | CGAACAATGTTTGTCGGGTTAGAG |
| shRNA oligonucleotides | |
| Human CACYBP | CATCAAGTTCCCACTGAGAAT |
| Human WWP2 | CCTCACCTACTTTCGCTTTAT |
| Mouse WWP2 | GCAGCACTTCAGCCAAAGATT |
| Human USP10 | GCCTCTCTTTAGTGGCTCTTT |
| siRNAs oligonucleotides | |
| Aedes albopictus Su(dx) | UUCGAAAGUUGUUUUCCUGUU |
| dsRNA oligonucleotides | |
| Aedes aegypti Su(dx)-Forward | TAATACGACTCACTATAGGGAGA ACAATACCACGGCTAATTTCAATG |
| Aedes aegypti Su(dx)-Reverse | TAATACGACTCACTATAGGGAGACCCAACCAGCAGGAAGAGG |

**Graphic Abstract**


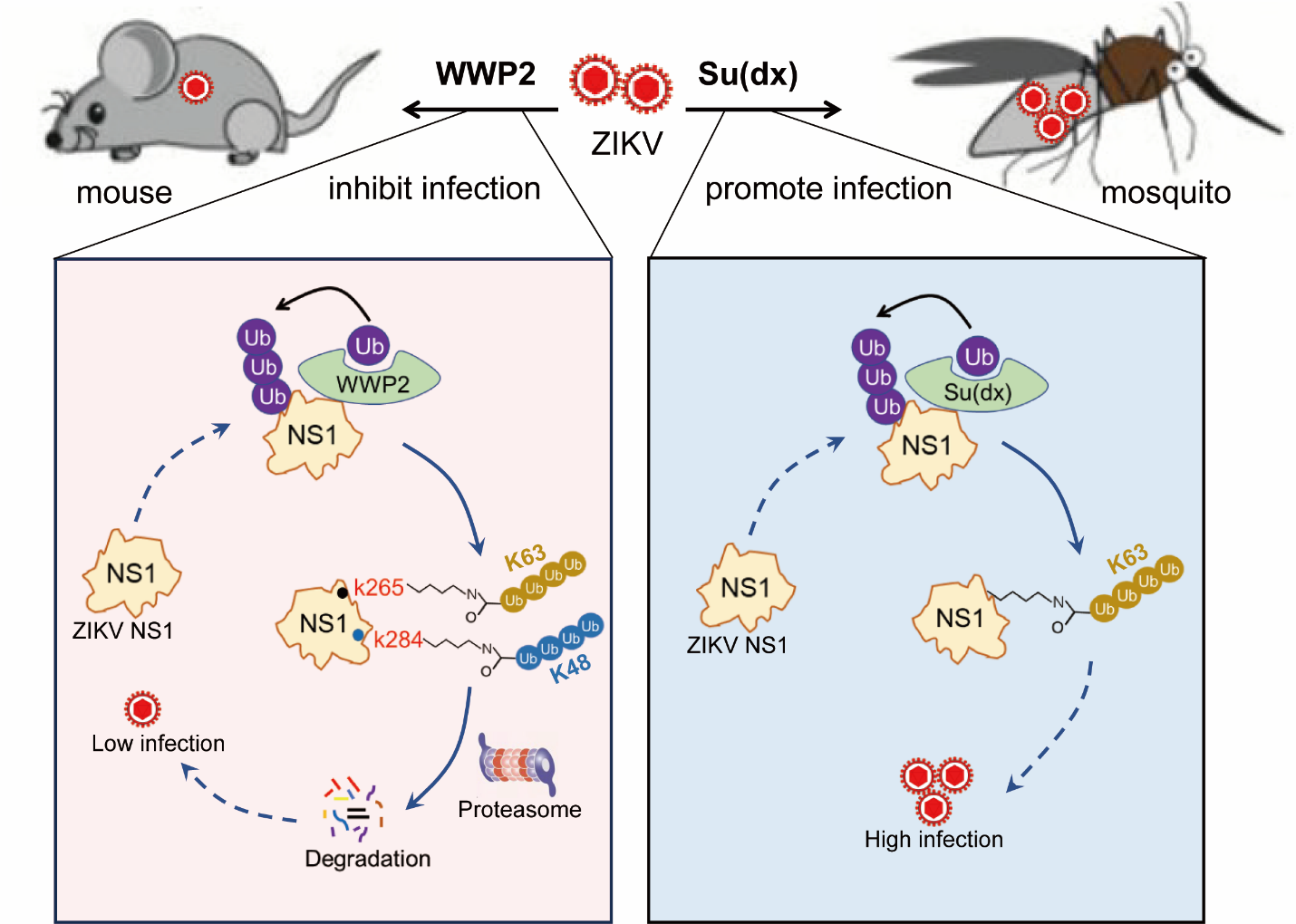

Supplement: Supplementary file 1 — Supporting Information [file ADVS-11-2408024-s001.docx]
